# Full Recovery of Polyimide Wastes Into High‐Value Products Through Break and Reconstruction of Imide Ring

**DOI:** 10.1002/advs.202414416

**Published:** 2025-02-12

**Authors:** Haodi Chen, Xuehui Liu, Shouqin Zhang, Shimei Xu, Teng Fu, Yu‐Zhong Wang

**Affiliations:** ^1^ State Key Laboratory of Polymer Materials Engineering Polymer Research Institute The Collaborative Innovation Center for Eco‐Friendly and Fire‐Safety Polymeric Materials (MoE) National Engineering Laboratory of Eco‐Friendly Polymeric Materials (Sichuan) Sichuan University Chengdu 610064 China; ^2^ College of Architecture and Environment The Collaborative Innovation Center for Eco‐Friendly and Fire‐Safety Polymeric Materials (MoE) National Engineering Laboratory of Eco‐Friendly Polymeric Materials (Sichuan) Sichuan University Chengdu 610064 China; ^3^ The Collaborative Innovation Center for Eco‐Friendly and Fire‐Safety Polymeric Materials (MoE) National Engineering Laboratory of Eco‐Friendly Polymeric Materials (Sichuan) College of Chemistry Sichuan University Chengdu 610064 China

**Keywords:** aminolysis, chemical recycling, hydrolysis, life cycle assessment, polyimide

## Abstract

Polyimide (PI) is a promising engineering plastic but difficult to recycle due to its aromatic heterocyclic structures, especially for thermosetting PI. Herein, an ingenious cascade pathway for high‐value and full recovery of thermosetting PI under mild conditions is presented. Specifically, the imide ring is first broken through aminolysis to degrade PI and is reconstructed under following hydrolysis to achieve excellent thermal stability in subsequent products. When recycling thermoplastic PI, the yield of 91% and 82% appears in diamines and polyhydroxylated compounds, respectively. As focusing on the recovery of thermosetting PI, aside from the efficient recovery of diamines, the crosslinked component is converted into hyperbranched flame retardant carbonizers for preparing copolyester, and it correspondingly achieves good flame retardant effect with an addition of merely 4 wt.%. The success of this work capitalizes on the unique structure and properties of the raw material, stimulating innovative pathways in the field of thermoset waste recovery and new material design.

## Graphical abstract

Effective degradation of PI to recycle diamine and polyhydroxy chemicals is successfully achieved via aminolysis‐coupled hydrolysis under mild conditions. Interestingly, the unique chemical structure of thermosetting PI allows the degraded product to be used as hyperbranched flame retardant carbonizer, resulting in excellent flame retardancy in polyester.

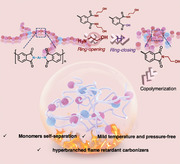

## 1 Introduction

Polyimide (PI), as a significant constituent of engineering plastics, behaves best with comprehensive properties such as exceptional mechanical properties, prominent chemical resistance, and excellent thermal stability, and is widely used in aerospace, flexible display, fireproof materials, and other scientific domains.^[^ ^1^, ^2^ ^]^ With the mass use of PI, the amount of its waste is increasing. However, most PI cannot turn into a processable molten state by heating,^[^ ^3^ ^]^ especially thermosetting PI with stable crosslinked structure. Worst yet, the stabilized aromatic heterocyclic rings embedded in the polymer backbone further increase the intractability of PI's recycling. Despite all this, there is an urgent need to promote the recycling of PI so as to control its manufacturing costs.

**Scheme 1:**
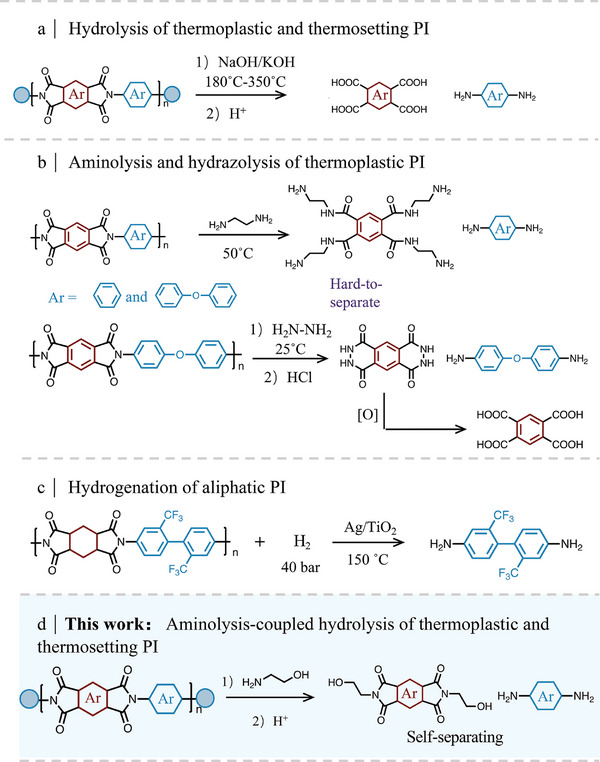
a) Hydrolysis of thermoplastic and thermosetting PI. b) Aminolysis and hydrazolysis of thermoplastic PI. c) Hydrogenation of aliphatic PI. d) Aminolysis‐coupled hydrolysis of thermoplastic and thermosetting PI in this work.

Chemical recovery has been identified as the most promising strategy for recycling value chemicals from plastic wastes.^[^ ^4^ ^]^ Currently, several efficient methods have been developed for recycling PI, such as hydrolysis, aminolysis, hydrazolysis, and hydrogenation. Among them, base‐catalyzed hydrolysis is one of the most common methods for recovering monomers from thermoplastic and thermosetting PI.^[^ ^5^, ^6^, ^7^, ^8^, ^9^ ^]^ NaOH or KOH as the representative catalyst during the hydrolysis reaction, can break the C─N bonds of PI to produce diamine and carboxylate products (**Scheme** **1**a). However, strong base‐mediated hydrolysis normally requires high temperature or extra pressure, potentially compromising the structure of diamine and diminishing the yield of monomers. Aminolysis and hydrazolysis are relatively mild, where thermoplastic PI can be degraded into diamide and other monomers at low temperature and atmospheric pressure (Scheme 1b).^[^ ^10^, ^11^ ^]^ Nevertheless, the utilization of explosive chemicals poses potential safety hazards, and the homogeneous system renders the cumbersome process of product separation and purification. Catalytic hydrogenation offers a green and cost‐effective solution for the cleavage of C─N bonds, but the cleavage efficiency in C─N bond of soluble aliphatic PI is pity even after heating at 150 °C for 15 h, resulting in a diamine yield of only 9% (Scheme 1c).^[^ ^12^ ^]^ To date, due to the chemical stability conferred by the aromatic heterocyclic structure, the recycling of PI is generally achieved either under harsh reaction conditions or with complex post‐processing, leading to a remarkable deterioration in monomer yield. What's more, the current recycling approaches are mainly applicable to thermoplastic PI and rarely to thermosetting PI. Unlike completely breaking the thermoplastic PI to recover the majority of monomers, the crosslinked structure in thermosetting PI is hard to break even under more severe conditions. In some cases, even if the crosslinked structure is destroyed, the degraded products are used in low value and inefficient due to their complex composition. Therefore, it is of great significance to develop recycling methods of thermosetting PI with mild reaction conditions to achieve direct, complete, and high‐value utilization of products.

Considering that the recovery of thermosetting polymers usually requires harsh reaction conditions to break their stable crosslinked structure, which is precisely the most difficult to achieve, but this unique structure shows great potential for designing new materials. Selectively inserting functional groups into the crosslinked structures and reconstructing them is key for achieving the above purpose. So, we present an aminolysis‐coupled hydrolysis pathway to recover high‐value‐added chemicals from both thermoplastic and thermosetting PI (Scheme 1d). Thermoplastic PI is used as the model compound, their C─N bond is first broken by ethanolamine (MEA) and forms four amide bonds and 4,4′‐oxydianiline (4,4′‐ODA). Following the hydrolysis route line, part of the amide bonds breaks to form carboxyl groups, which form an imide ring via dehydrating with their ortho‐amide bonds, thus, N, N′‐(2‐hydroxyethyl) pyromelitimide (NNHPI) can directly self‐separate from the reaction solution. More intriguingly, this methodology performs well in recycling thermosetting PI, value‐added diamine and the other parts containing crosslinked structures are successfully collected in high quality. Compared with the traditional single‐pathway degradation focusing solely on recovering monomers, this method has two significant advantages: first, the selective breakage of C─N bond under mild conditions to avoid the damage of sensitive monomers; and second, the utilization of unique crosslinked structure in thermosetting PI and the reconstruction of functional groups inserted to achieve the high‐value utilization of all the degraded products. In addition, the results of the life cycle assessment (LCA) confirm that this recycling pathway is more eco‐friendly than traditional hydrolysis or natural degradation. Our work here not only realizes high‐efficient and complete recycling of PI under mild conditions but also provides an ingenious pathway for the design of functional chemicals that would be unattainable through conventional chemical synthesis methods.

## 2 Results and Discussion

### 2.1 The Degradation of Thermoplastic PI

Kapton film, as a type of thermoplastic PI, exhibits the typical structure and property characteristics of the PI family (Figures S1 and S2, Supporting Information). So Kapton film is chosen as the model compound and degraded in diverse amines (Table S1, Supporting Information). Since the reactions are preferably set at atmospheric pressure, the aminolysis temperature was set according to the boiling point of diverse amines. In diethylamine and N‐butylamine, probably due to the lower temperature, the degradation rate of Kapton film is lower even with a reaction time of 8 h. When amine with a high boiling point was used, the degradation rate of Kapton film increased with the reaction temperature. In particular, Kapton film was completely degraded in ethylenediamine and MEA, and the yield of 4,4′‐ODA recovered from both reaction systems is high. However, only a few amide products were observed in ethylenediamine and 1,3‐diaminopropane, possibly due to two amino‐terminated groups in diamine resulting in the formation of multimers (Figures S3 and S4, Supporting Information). In MEA, the amide products are THPA with an NMR yield of 84%. Based on the comprehensive consideration of reaction safety, product properties and yield, MEA was used in the recycling of Kapton film. Even so, actually, the separation of THPA and 4,4′‐ODA is still challenging.

Therefore, an aminolysis‐coupled hydrolysis was designed to efficiently degrade Kapton film and isolate the degraded product in situ. The obtained degraded products are 4,4′‐ODA and NNHPI. Note that the aminolysis process affects the yield of 4,4′‐ODA and NNHPI simultaneously (Table S2, Supporting Information). When the aminolysis temperature increases from 100 to 120 °C, the yield of 4,4′‐ODA increases from 34% to 91%, the yield of NNHPI increases from 24% to 82%. Further raising the aminolysis temperature to 140 °C, the yields of 4,4′‐ODA and NNHPI drop slightly. Extending aminolysis time from 1 to 3 h significantly increases the yield of 4,4′‐ODA from 39% to 91%, and the yield of NNHPI from 28% to 82%, whereas prolonged aminolysis time up to 5 h results in a slight decrease in their yield (85% in 4,4′‐ODA and 74% in NNHPI) due to the high activity and oxidation prone of monomers.^[^ ^13^ ^]^ To confirm this, the degradation of Kapton film under N_2_ was attempted. Compared to experiments performed in the air (Table S2, Supporting Information, entry 4), the yield of 4,4′‐ODA increases from 85% to 91%, and the yield of NNHPI increases from 74% to 76% after N_2_ protection (Table S2, Supporting Information, entry 13). In hydrolysis, the yield of 4,4′‐ODA is in the range of 90%–96% and not significantly impacted by hydrolysis temperatures, but the yield of NNHPI varies significantly with the hydrolysis temperature. At 25 °C, NNHPI cannot be obtained. By increasing the temperature to 60 °C, the yield of NNHPI is 7%. The highest yield of NNHPI is 82% at 100 °C. Similar to the prolongation of the aminolysis time, prolonging the hydrolysis time also decreases the monomers yield, especially for electron‐rich and less conjugative 4,4′‐ODA. Besides, Kapton film cannot be directly degraded in an HCl solution (Figure S5, Supporting Information). The above results disclose that the C─N bond of Kapton film is completely destroyed in aminolysis and hydrolysis only promotes the formation of NNHPI.

**Figure 1:**
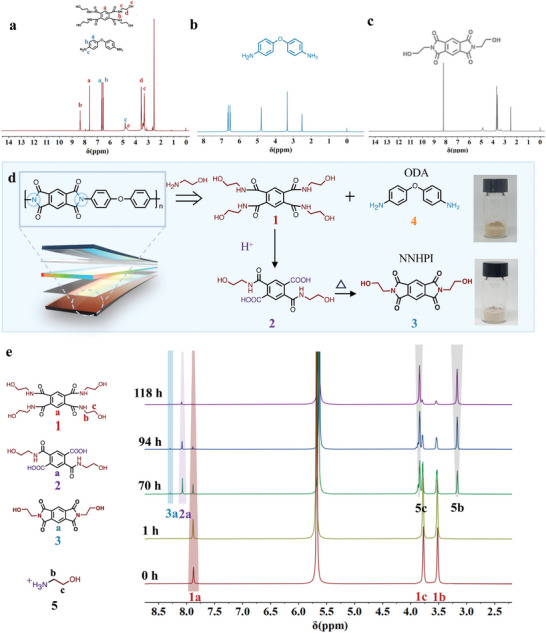
The degradation of Kapton film. a) The ^1^H‐NMR spectrum of aminolysis products, b) isolated 4,4′‐ODA, and c) NNHPI. d) The proposed degradation mechanism of Kapton film by aminolysis‐coupled hydrolysis. e) Time‐stacked ^1^H‐NMR spectra of the reaction of THPA at DCl.

To provide insights into the underlying mechanism of this recycling process, the chemical structure of aminolysis products was studied. As shown in **Figure** **1**a and S6 (Supporting Information), no NNHPI is detected, instead, it is N, N′, N″, N‴‐1,2,4,5‐tetra pyromellitamide (THPA) accompanied by 4,4′‐ODA in the aminolysis products. Besides, the presence of 4,4′‐ODA suggests that the diamine is formed in the aminolysis. With the aim of effectuating the segregation of monomers, HCl was added to protonate 4,4′‐ODA to form aqueous ammonium salt that can be obtained again after neutralization (Figure 1b; Figures S7 and S8, Supporting Information). Notably, the THPA disappeared and NNHPI precipitated after the addition of acid, realizing the separation of NNHPI from ammonium salt (Figure 1c; Figure S9, Supporting Information). A series of methods have been previously proposed to synthesize THPA through NNHPI,^[^ ^14^ ^]^ however, it remains unclear how to convert THPA to NNHPI. Thus, we pursued a series of control experiments to understand the conversion mechanism of THPA and NNHPI. To rule out the possibility of directly converting NNHPI to THPA by heating, THPA was heated at 170 °C (boiling point of MEA). Apparently, no NNHPI was detected, revealing that HCl may participate in the formation of the imide ring (Figure S10, Supporting Information). To confirm this, the aminolysis products of Kapton film were hydrolyzed under neutral and alkaline conditions, respectively. The NNHPI exclusively occurred under acidic conditions, while it was not produced under neutral and alkaline conditions (Table S3 and Figures S11 and S12, Supporting Information). In that case, we envision that THPA is hydrolyzed to intermediate (**2**) by HCl, in which the carboxyl group reacts with ortho‐amide via intramolecular dehydration to produce NNHPI (Figure 1d).

Further, time‐stacked proton nuclear magnetic resonance (^1^H‐NMR) was used to monitor the conversion of THPA in deuterium chloride (DCl) at room temperature. As shown in Figure 1e, only THPA is observed in the beginning, then intermediate (**2**) and protonated MEA (**5**) appear as the reaction time is extended. Subsequently, NNHPI began to appear. Finally, all intermediate (**2**) was converted to NNHPI (**3**) and precipitated in the nuclear magnetic tube (Figure S13, Supporting Information). The above results substantiate the aforementioned hypothesis.

### 2.2 The Degradation of Thermosetting PI

**Figure 2:**
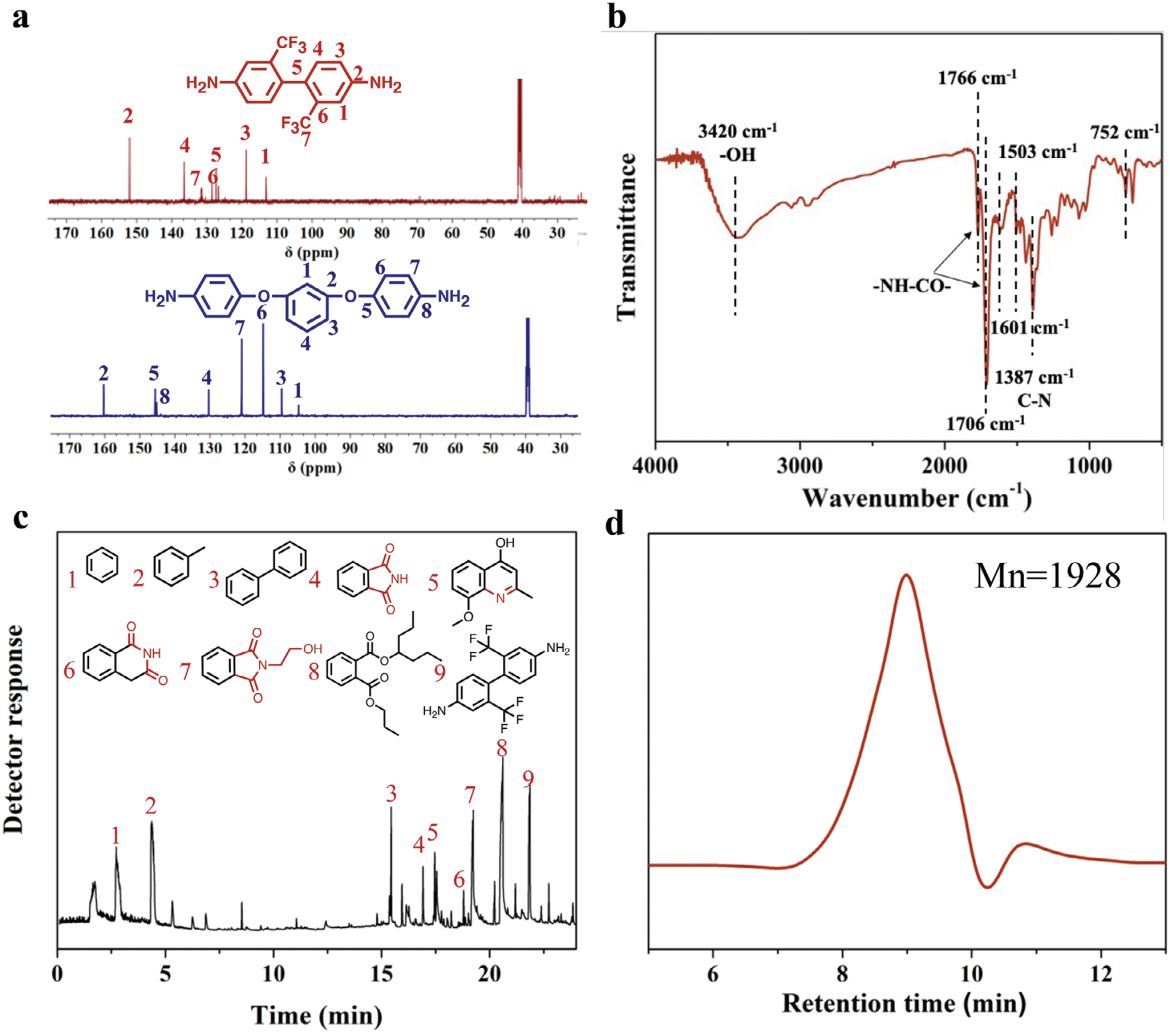
The chemical structure of degraded products from PETI. a) ^13^C‐NMR spectra of recovered TFMB and 1,3,4‐APB. b) FTIR spectrum, c) total ion chromatograms, and d) GPC trace of DPETI.

After making processes in thermoplastic PI, we turned our attention to thermosetting PI with the three‐dimensional crosslinked network. Pleasingly, this strategy was compatible with thermosetting phenylethynyl end‐capped polyimide (PETI), a mixture consisting of 2,2′‐bis(trifluoromethyl)benzidine (TFMB) and 1,3‐bis(4‐aminophenoxy) benzene (1,3,4‐APB) with a ratio of 1:1 was recovered after aminolysis‐coupled hydrolysis (**Figure** **2**a; Figure S14, S15 and S16, Supporting Information). Further, the effects of aminolysis and hydrolysis conditions on the yield of diamine were explored respectively (Table S4, Supporting Information). In analogy to thermoplastic PI, the longer aminolysis time led to higher diamine yield. However, the yield of diamine also increased with the increase in hydrolysis temperature, time, and HCl concentration. This is different from that of thermoplastic PI, aminolysis and hydrolysis play together in the cleavage of the C─N bond of thermosetting PI. In the optimized reaction conditions, the yield of diamine is up to 61%, higher than that of hydrolysis work.^[^ ^9^ ^]^

Similar to the degraded products of Kapton film, new aromatic heterocyclic rings and hydroxyl groups may occur in the degraded products of PETI after hydrolysis (DPETI). In the Fourier transform infrared (FT‐IR) spectrum of DPETI, the peaks attributed to the imide ring appear at 1766, 1706, and 1387 cm^−1^, and the peak attributed to hydroxyl group appear at 3420 cm^−1^ (Figure 2b). Moreover, 2‐(2‐hydroxyethyl)isoindoline‐1,3‐dione (**7**) is also found in the pyrolysis‐gas chromatography‐mass spectrometry (Py‐GC‐MS) of DPETI (Figure 2c). In addition, the weight average molecular weight of DPETI is 1928 with a PDI of 1.99 (Figure 2d; Table S5, Supporting Information).

**Figure 3:**
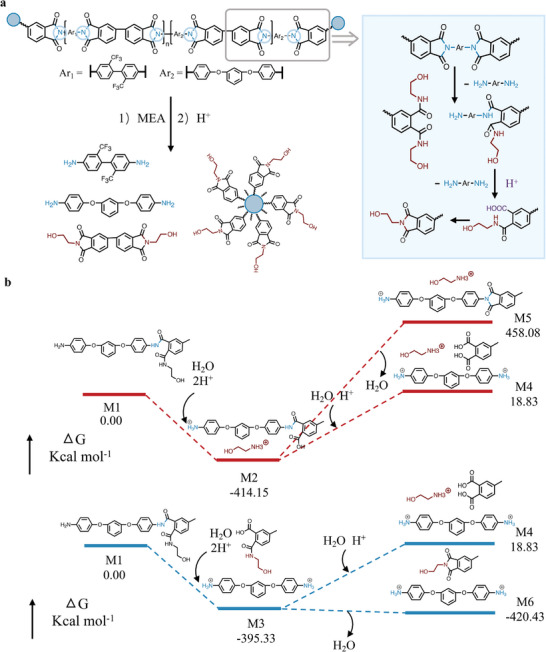
a) The proposed degradation mechanism of PETI by aminolysis‐coupled hydrolysis. b) Gibbs free energy for the hydrolysis of PETI.

Based on the above results, we propose the degradation mechanism of PETI by aminolysis‐coupled hydrolysis (**Figure** **3**a). Starting from the disruption of the C─N bond by MEA, the partial diamine is produced due to the breakage of four C─N bonds (Figure S17, Supporting Information).^[^ ^15^ ^]^ There are also cases where only one, two, or three C─N bonds are broken. Thus, some amide bonds are formed after aminolysis, partial of which can be broken through hydrolysis to generate the other part diamine. In other words, the obtained diamine originates from two paths of C─N cleavage in aminolysis and amide cleavage in hydrolysis. Meanwhile, dehydration occurs between the carboxyl group and amide bond to conduct the imide ring‐closing. However, when only one imide ring or C─N bond is broken, diamine cannot be recovered because H^+^ is hard to break the other stable imide ring under these conditions.

In order to obtain more insight into the mechanism of hydrolysis, density functional theory (DFT) calculations were performed (Figure 3b). Aminolysis products are simplified to M1(0.00 Kcal mol^−1^) and used as the representative sample to simulate hydrolysis. Under acidic conditions, the amide bonds of M1 may be broken in two ways, the cleavage on the aliphatic chain is to form M2, while the cleavage on the aromatic chain is to form M3. The Gibbs free energies of M2 (−414.15 Kcal mol^−1^) and M3 (−395.33 Kcal mol^−1^) are relatively close, proving the coexistence of these two pathways. In the further reaction, M2 may be produced as M4 by the breaking of the amide bonds or as M5 by dehydration. Similarly, the cleavage of the amide bonds in M3 leads to the formation of M4; alternatively, M3 can be converted to M6 through a dehydration process. However, the energy values of M4 (18.83 Kcal mol^−1^) or M5 (458.08 Kcal mol^−1^) are higher than M2, indicating the difficulties in both hydrolysis and dehydration of M2. In addition, the higher energy of M4 relative to M3 suggests that the cleavage of the amide bond in M3 is challenging. While the conversion of M3 to M6 is viable because the energy from M3 to M6 drops into the lowest state of −420.43 kcal mol^−1^. This explains that M3 is prone to dehydrate between the carboxyl group and amide bond rather than the disruption of the second amide bond.

### 2.3 The Reutilization of DPETI

**Figure 4:**
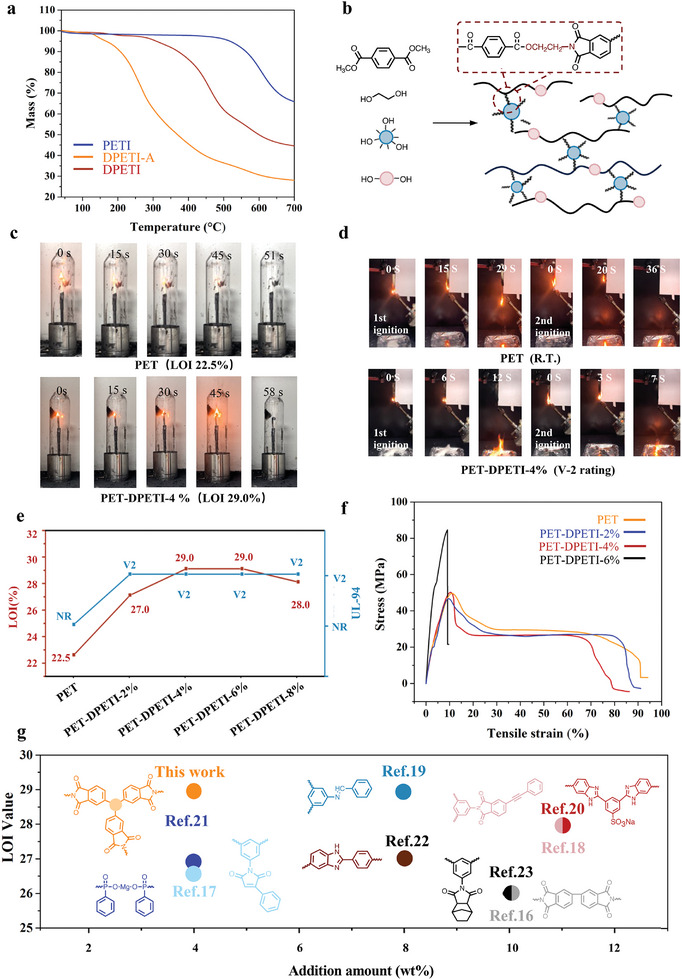
Fire safety of copolyesters. a) TGA curves of PETI, DPETI‐A, and DPETI in N_2_ atmosphere. b) Schematic diagram of copolyester preparation. c) Combustion processes of PET and PET‐DPETI‐4% during the LOI test. d) Combustion processes of PET and PET‐DPETI‐4% during the UL‐94 test. e) Comparison of the flame retardancy of copolyester with different content of DPETI. f) Stress–strain curves of PET and copolyesters. g) Comparison of the LOI value of copolyester with the previously reported works.

Considering the excellent thermal stability and high residual carbon from thermosetting PI, DPETI containing the crosslinked structure and aromatic heterocyclic rings may be used as hyperbranched flame retardant carbonizers. As shown in **Figure** **4**a, the initial decomposition temperature (T_5%_), the maximum decomposition temperature (T_max_) and char residue (CR) of degraded products of PETI after aminolysis (DPETI‐A) significant decline compared to PETI. After regeneration of the imide ring in hydrolysis, T_max_ of DPETI increases to 460 °C, and the  CR is 45 wt.% at 700 °C, exhibiting potential as flame retardant carbonizers. Due to the abundance of hydroxyl groups, DPETI was used to synthesize flame‐retardant polyethylene terephthalate (PET) copolyester (Figure 4b). Solubility tests verify that the slight crosslinked structure appears in the copolyester, it may be beneficial for copolyester in terms of the anti‐dripping performance and mechanical properties (Figure S18, Supporting Information). The thermal behavior of copolyesters was analyzed (Figures S19 and S20 and Table S6, Supporting Information). T_5%_ and T_max_ of copolyesters are ≈380 and 425 °C, respectively, which are similar to pure PET. But the CR at 700 °C of PET‐DPETI‐6% increases to 19.7 wt.% compared to pure PET, suggesting the DPETI facilitates the char formation. The limiting oxygen index (LOI) and vertical burning test (UL‐94) were used to evaluate the flammability of copolyesters (Figure 4c–e). PET is intrinsically flammable with a low LOI value of 22.5, while the LOI value of copolyester increases obviously. Only with the addition of 2 wt.% DPETI, the LOI value of copolyester increases to 27. Further increasing the addition to 4 wt.%, the LOI value of copolyester is 29. Compared with the previously reported works,^[^ ^16^, ^17^, ^18^, ^19^, ^20^, ^21^, ^22^, ^23^ ^]^ a small amount addition of DPETI performs well in LOI value of copolyester (Figure 4g). This value decreases slightly at 8 wt.% addition due to the decline of polymerization (Table S7, Supporting Information). Violent burning droplets that can lead to fire spread and secondary burning need to be a concern. As shown in Figure 4d, continuous droplets can be observed in the burning of PET, which is evaluated as no rating. As expected, the anti‐dripping and self‐extinguishing performances of copolyester are enhanced because of its slight crosslinked structure, exhibiting low dripping hazards and passing V‐2 level.

To the best of our knowledge, it is very difficult for flame retardant polyesters to balance their flame retardancy and mechanical properties. So, the effect of DPETI on the mechanical properties of copolyester was investigated. PET‐DPETI‐2% and PET‐DPETI‐4% exhibit the same mechanical behavior as pure PET, with a tensile strength of ≈50 MPa and the elongation at break of 80%. When the addition of DPETI is increased to 6 wt%, the tensile strength of the copolyester is up to 84 MPa, accompanied by a certain loss of toughness for its higher crosslinked degree (Figure 4f).

### 2.4 Environmental Effect

**Figure 5:**
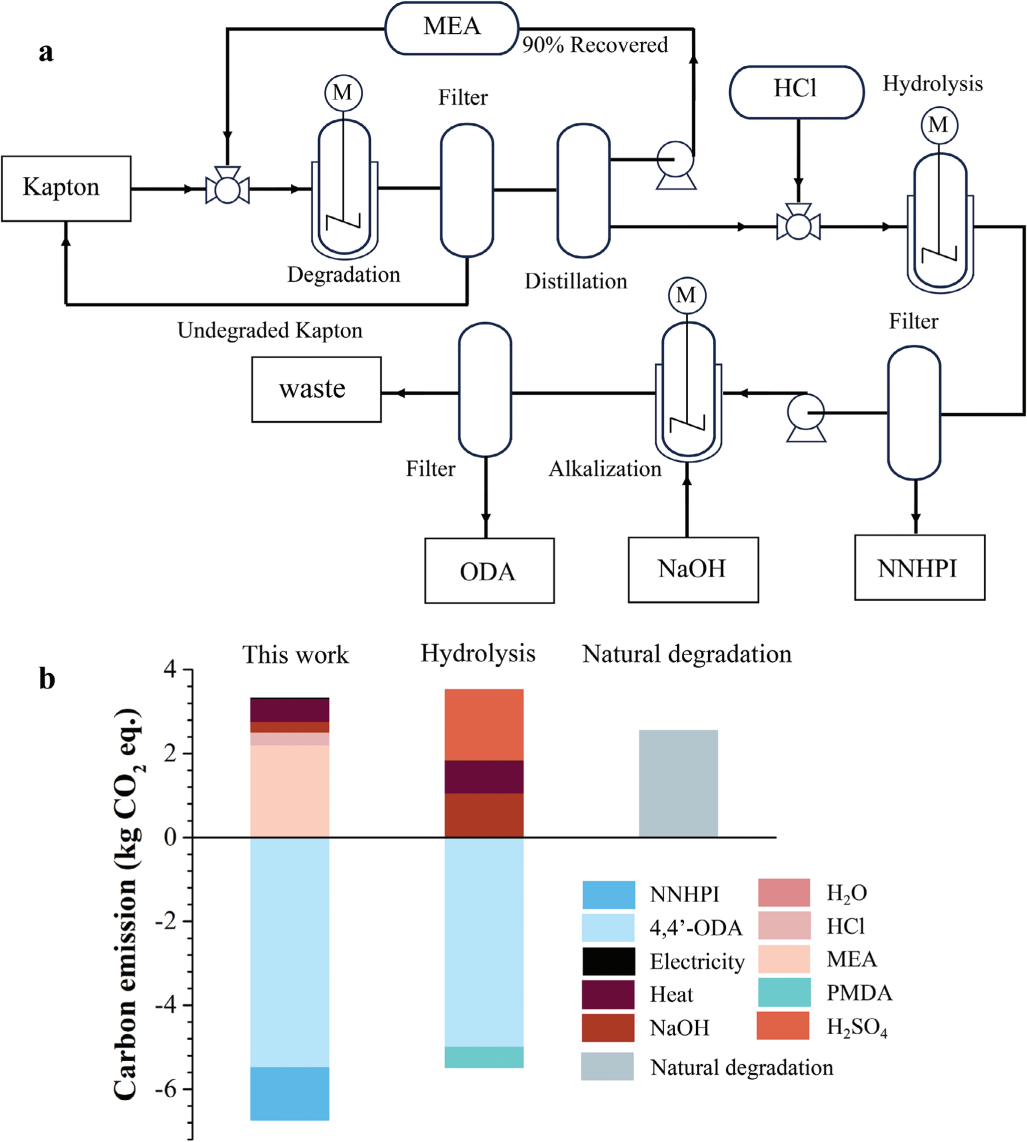
Assessment of carbon footprints. a) Chemical recycling model of Kapton film. b) Comparison of the carbon emissions of this work with traditional hydrolysis and natural degradation.

LCA (life cycle assessment) is used to assess the environmental effects of this recovery strategy. **Figure** **5**a shows the environmental impacts assessment model for the entire recycling process of Kapton film, where the carbon emissions are expressed in terms of carbon dioxide equivalent (CO_2_‐eq) per kg of waste Kapton film through the method of the Institute of Environmental Sciences (CML2001 baseline).^[^ ^24^ ^]^ In the entire aminolysis‐coupled hydrolysis recycling process, the carbon emissions of inputs are 3.3205 kg CO_2_ equiv, while that of the outputs are −6.7266 kg CO_2_ equiv (Figure 5b; Table S8, Supporting Information). Thus, the overall LCA presents that the total carbon emissions of this recycling are ‐3.4061 kg CO_2_ equiv, realizing negative carbon emissions. While, the carbon emission of 1 unit of Kapton film was 2.5336 kg CO_2_ equiv after full degradation to CO_2_ in the natural environment. Compared to other chemical recovery methods, such as conventional hydrolysis, in which only 1.9627 kg CO_2_ equiv is saved due to higher energy consumption and relatively low product yield, and this value occupies 58% of our method (Table S9, Supporting Information). Therefore, this recovery strategy of PI is promising circular economy and carbon.

## 3 Conclusion

In conclusion, we have successfully converted waste thermosetting PI into diamine and hyperbranched flame retardant carbonizers via aminolysis‐coupled hydrolysis, realizing high‐value and full utilization of degraded products. During the degradation, aminolysis achieves the cleavage of the imide ring and the formation of diamine and amide. Then acid protonates diamine and breaks amide bonds to form carboxyl groups, followed by the formation of an imide ring through dehydration between the ortho‐amide bond and carboxyl group. For thermoplastic PI, the yields of diamine and polyhydroxyl chemicals are up to 91% and 82%, respectively. While for thermosetting PI, the yield of diamine is 60%, showing considerable improvements versus traditional hydrolysis. Most interestingly, we effectively reuse the remaining thermosetting PI parts except diamine through this ingenious recycling path in mild condition. This remaining thermosetting PI parts containing the crosslinked structure, aromatic heterocyclic rings and abundant hydroxyl groups can be directly used to prepare flame‐retardant copolyesters, which have passed V‐2 level in UV‐94 test and the LOI value of 29 with only 4 wt.% addition. Moreover, the mechanical properties of the copolyester are not affected. In addition, this recycling approach realizes negative carbon emissions with a CO_2_ equiv of −3.4061 kg. Compared with conventional degradation routes, this cascade reaction pathway shows better overall benefits in terms of reaction conditions, reutilization of degraded products and carbon emissions, strengthening new recycling understandings of other plastic wastes.

## Conflict of Interest

The authors declare no conflict of interest.

## Supporting information



Supporting Information

## Data Availability

The data that support the findings of this study are available from the corresponding author upon reasonable request.

## References

[1] I. Gouzman , E. Grossman , R. Verker , N. Atar , A. Bolker , N. Eliaz , Adv. Mater. 2019, 31, 1807738. 10.1002/adma.201807738 30803081

[2] B. Wan , X. Dong , X. Yang , J. Wang , M.‐S. Zheng , Z.‐M. Dang , G. Chen , J.‐W. Zha , Adv. Mater. 2023, 35, 2301185. 10.1002/adma.202301185 36906511

[3] X. Lei , Y. Jin , H. Sun , W. Zhang , J. Mater. Chem. A 2017, 5, 21140.

[4] X. Liu , S. Xu , F. Zhang , X. Wang , Y.‐Z. Wang , Acta Polym. Sin. 2022, 53, 1005.

[5] L. E. Stephans , A. Myles , R. R. Thomas , Langmuir 2000, 16, 4706.

[6] H. Okumura , T. Takahagi , N. Nagai , S. Shingubara , J. Polym. Sci. Polym. Phys. 2003, 41, 2071.

[7] F. Huang , Y. Huang , Z. Pan , Ind. Eng. Chem. Res. 2012, 51, 7001.

[8] T. Honma , T. Sato , J. Supercrit. Fluids 2020, 166, 105037.

[9] Q. Zhao , X. Li , Z. Tian , H. Ma , X. Hou , Y. Wang , Y. Wang , Composites, Part B 2022, 231, 109595.

[10] Z. Tian , Q. Zhao , X. Liu , Y. Wang , J. Zhao , Y. Wang , X. Hou , ACS Sustain. Chem. Eng. 2023, 11, 11590.

[11] R. A. Dine‐Hart , D. B. V. Parker , W. W. Wright , Br. Polym. J. 1971, 3, 226.

[12] X. Liu , C. Wu , P. Bai , Y. Miao , Y. Hu , Y. Xie , Org. Lett. 2023, 25, 3066. 37088958 10.1021/acs.orglett.3c00887

[13] H. Zhao , X. Zhao , J. Zhang , S. Anandita , W. Liu , S. W. Koh , S. Yu , C. Li , Z. Chen , R. Xu , Z. Zou , W. Tu , H. Li , Adv. Energy Mater. 2024, 14, 2400037.

[14] J. E. A. Webb , M. J. Crossley , P. Turner , P. Thordarson , J. Am. Chem. Soc. 2007, 129, 7155. 17497782 10.1021/ja0713781

[15] H. Chen , X. Liu , Q. He , S. Zhang , S. Xu , Y. Z. Wang , Adv. Mater. 2023, 36, 2310779.

[16] Z.‐Z. Wu , Y.‐P. Ni , T. Fu , B.‐W. Liu , W. S. Wu , L. Chen , X.‐L. Wang , Y.‐Z. Wang , Polym. Degrad. Stab. 2018, 155, 162.

[17] X. Dong , L. Chen , R.‐T. Duan , Y.‐Z. Wang , Polym. Chem. 2016, 7, 2698.

[18] L. Chen , H.‐B. Zhao , Y.‐P. Ni , T. Fu , W.‐S. Wu , X.‐L. Wang , Y.‐Z. Wang , J. Mater. Chem. A 2019, 7, 17037.

[19] J.‐N. Wu , L. Chen , T. Fu , H.‐B. Zhao , D.‐M. Guo , X.‐L. Wang , Y.‐Z. Wang , Chem. Eng. J. 2018, 336, 622.

[20] W.‐S. Wu , P.‐H. Duan , Y.‐L. Wang , L. Chen , X.‐L. Wang , Y.‐Z. Wang , Sci. China Mater. 2021, 64, 2067.

[21] X. Li , J. Guan , W. Zeng , H. Li , J. Shi , N. Wen , Z. Yang , Z. Lei , Eur. Polym. J. 2022, 171, 111174.

[22] Y.‐P. Ni , Q.‐T. Li , L. Chen , W.‐S. Wu , Z.‐H. Qin , Y. Zhang , L. Chen , X.‐L. Wang , Y.‐Z. Wang , Chem. Eng. J. 2019, 374, 694.

[23] X. Dong , R.‐T. Duan , Y.‐P. Ni , Z.‐J. Cao , L. Chen , Y.‐Z. Wang , Polym. Degrad. Stab. 2017, 146, 105.

[24] J. Guinée , Int. J. Life Cycle Assess. 2001, 6, 255.

