# Supplementary material for: Full Recovery of Polyimide Wastes Into High‐Value Products Through Break and Reconstruction of Imide Ring
Source: Adv Sci (Weinh). 2025 Feb 12;12(13):2414416. doi: 10.1002/advs.202414416 (PMC11967771; doi:10.1002/advs.202414416)
Supplement: Supplementary file 1 — Supporting Information [file ADVS-12-2414416-s001.docx]

**Full recovery of polyimide wastes into high-value products through break and reconstruction of imide ring**

*Haodi Chen^1^, Xuehui Liu^2^*, Shouqin Zhang^3^, Shimei Xu^3^, Teng Fu^1^, Yu-Zhong Wang^3^**

^1^State Key Laboratory of Polymer Materials Engineering, Polymer Research Institute, The Collaborative Innovation Center for Eco-Friendly and Fire-Safety Polymeric Materials (MoE), National Engineering Laboratory of Eco-Friendly Polymeric Materials (Sichuan), Sichuan University, Chengdu 610064, China.

^2^College of Architecture and Environment, The Collaborative Innovation Center for Eco-Friendly and Fire-Safety Polymeric Materials (MoE), National Engineering Laboratory of Eco-Friendly Polymeric Materials (Sichuan), Sichuan University, Chengdu 610064, China.

^3^The Collaborative Innovation Center for Eco-Friendly and Fire-Safety Polymeric Materials (MoE), National Engineering Laboratory of Eco-Friendly Polymeric Materials (Sichuan), College of Chemistry, Sichuan University, Chengdu 610064, China.

E-mail: liuxuehui@scu.edu.cn, [yzwang@scu.edu.cn](mailto:yzwang@scu.edu.cn)

| **Table of Contents** | **pages** |
| --- | --- |
| Supplementary Method | S3 |
| Supporting Figures and Tables | S18 |
| Supplementary References | S49 |
|  |  |

**Supplementary Method**

**Materials**

Phenylethynyl end-capped polyimide oligomers were provided by AECC Beijing Institute of Aeronautical Materials, Beijing, China. Kapton film was purchased from Shenzhen yingshida Plastic Materials Co., Ltd. Ethanolamine (MEA, 98.0%-100.5%), ethylenediamine, HCl, H_2_SO_4_ and NaOH were purchased from Chengdu Kelong Chemical Reagent Company (China). Dimethyl sulfoxide-d_6_ (DMSO‑d_6_, 99.9 atom % D), DCl (DCl 20% in D_2_O, 99.5 atom % D) and water-d_2_ (D_2_O, 99.9 atom % D) were purchased from Qingdao Teng Long Microwave Technology Co., Ltd. Pyromellitic dianhydride (PMDA), 4,4’-Oxydianiline (4,4’-ODA), 2,2’-Bis(trifluoromethyl)benzidine (TFMB), 1,3-Bis(4-aminophenoxy)benzene (1,3,4-APB), diethylamine, N-butylamine, 1,3-diaminopropane and 1,1,2,2-tetrachloroethane were purchased from Titan. All chemicals were directly used as received without further purification.

**Experimental procedures**

**The synthesis of thermosetting PI**

Phenylethynyl end-capped polyimide oligomers were put into a metal mold and cured at 360 ℃ in an oven. After 2 h, the curd phenylethynyl end-capped polyimide (PETI) was obtained.

**The synthesis of pure N,N’-(2-hydroxyethyl) pyromelitimide (NNHPI)**

NNHPI was synthesized according to the literature method[1]. 1.74 g PMAD and 1.00 g MEA were added to 5 mL of N, N-dimethylformamide. After refluxing for 2 h, the reaction solution was poured into 100 mL of water to precipitate pearl white solid.

^1^H-NMR (400 MHz, DMSO-d_6_): δ= 3.63 (t, 2H, 5.29), 3.71 (t, 2H), 4.88 (s, 1H), 8.2 (s, 1H).

^13^C-NMR (101 MHz, DMSO-d_6_): δ= 166.89, 137.49, 117.48, 58.27, 41.39.

**The synthesis of pure N,N’,N’’,N’’-1,2,4,5-Tetra(hydroxyethyl) Pyromellitamide (THPA)**

According to the literature[1], NNHPI (0.10 g, 0.3 mmol) was suspended in ethanol (6 mL) at 60 °C, and then MEA(0.17 g, 2.8 mmol) was added. After 1 h, the reaction solution was filtered. The collected filter cake was washed with ethanol to obtain white powder.

^1^H-NMR(400 MHz, DMSO-d_6_)：δ= 3.62 (q, 2H, 5.81Hz), 3.70 (t, 2H, 5.32 Hz), 4.91 (t, 1H, 6.06 Hz), 8.19 (s, 1H).

**The traditional hydrolysis of thermoplastic PI**

According to the literature[2], 3 g Kapton, 2.5 g NaOH and 15 mL H_2_O were added into a round-bottom flask equipped with a magnetic stir bar. Subsequently, the reaction was performed at 100 ℃ for 8 h, and then 4,4’-ODA was collected by filtration. After adding H_2_SO_4_, white solids were precipitated from the filtrate, which then was heated in 50 wt% H_2_SO_4_ to form 1, 2, 4, 5-Benzenetetracarboxylic anhydride (PMDA).

**Degradation of thermoplastic PI** **via aminolysis-coupled hydrolysis**

2 g Kapton film (10 mm × 20 mm × 0.1 mm) and 20 mL MEA were added into a round-bottom flask equipped with a magnetic stir bar. Subsequently, the reaction was performed at 120 ℃ for 1-3 h. The resulting mixture was concentrated by distillation under reduced pressure. Then, 15 mL HCl was added and reacted with the mixture at 25-100 ℃ for 1-3 h. After that, NNHPI was collected by filtration. The diamines were precipitated from the filtrate after adding NaOH.

4,4’-Oxydianiline (4,4’-ODA):

^1^H-NMR(400 MHz, DMSO-d_6_)：δ= 4.78 (s, 1H), 6.52 (m, 1H, 8.67 Hz), 6.68(m, 1H, 8.67 Hz).

^13^C-NMR(101 MHz, DMSO-d_6_)：δ= 148.87, 144.56, 119.40, 115.24.

**Degradation of thermosetting PI** **via aminolysis-coupled hydrolysis**

2 g PETI and 20 mL MEA were added into a round-bottom flask equipped with a magnetic stir bar. Subsequently, the reaction was performed at 120 ℃ for 0.5-6 h. The resulting mixture was concentrated by distillation under reduced pressure. Then, 15 mL HCl was added and reacted with the mixture at 25-100 ℃ for 0.5-6 h. After that, DPETI was collected by filtration. The diamines were precipitated from the filtrate after adding NaOH.

2,2’-Bis(trifluoromethyl)benzidine (TFMB):

^13^C-NMR (151 MHz, DMSO-d_6_)：δ= 148.73, 133.51, 128.68 (q, J_C−F_ = 30.0 Hz), 124.81 (q, J_C−F_ = 275.9 Hz), 124.56, 116.24, 110.67.

^1^H-NMR (400 MHz, DMSO-d_6_)：δ=5.55 (s, 1H), 6.73 (dd, J=8.3, 2.4Hz, 1H), 6.88 (d, J-8.2Hz), 6.91 (d, J=2.4Hz, 1H).

1,3-Bis(4-aminophenoxy)benzene (1, 3, 4-APB) :

^1^H-NMR(400 MHz, DMSO-d_6_)：δ=5.0 (s, 4H), 6.34(t, J=2.4Hz, 1H), 6.45 (dd, J=8.2,2.4 Hz, 2H), 6.59(m, 4H), 6.75(m, 4H), 7.18(t, 8.2Hz,1H).

^13^C-NMR (151 MHz, DMSO-d_6_)：δ= 160.76, 146.11, 145.52, 130.78, 121.51, 115.27, 109.97, 105.14.

**Preparation of PET and copolyesters**

The preparation of copolyester with 2-8 wt% DPETI was described as follows. First, DMT (58.2g, 0.30 mol), EG (40.9 g, 0.66 mol), BPDI (1.25-6 g) and tetrabutyltitanate solution (0.2 mL) were added into a 250 mL polymerization bottle equipped with the mechanical stirring. The mixture was heated to 180 ℃ for 3 h under nitrogen atmosphere for transesterfication, while methanol was taken away and collected by nitrogen flow. Then, the mixture was heated to 250 ℃ under the pressure of 80-100 Pa. After the reaction for 3 h, the whole system was cooled to room temperature under vacuum and the copolyesters was obtained. Pure PET was also synthesized by the same procedures.

**Characterization**

Fourier transform infrared (FTIR) spectra were collected on a Nicolet 6700 spectrophotometer (ThermoFisher Scientific, USA) at ambient temperature. Liquid-state NMR spectra were obtained with an Avance II-400 MHz spectrometer (Bruker, Switzerland) using DMSO‑d_6_ as solvents. Time-stacked ^1^H-NMR spectra were obtained with an Avance II-500 MHz spectrometer (Bruker, Switzerland) using 0.3 mL DCl and 0.3 mL D_2_O as solvents. Gas chromatography-mass spectrometry (GC-MS) was carried out on GCMS-QP2010 Plus (SHIMADZU, Japan). Liquid Chromatograph Mass Spectrometer (LC-MS) was carried out on Waters ZQ2000 (Waters, USA) instrument. Molecular weights of the degraded products were determined by gel permeation chromatography (GPC) on a PL gel MINED-BLS separation system (Agilent, USA). Molecular weights of the soluble part of copolyester were determined by GPC on waters 2695 (Waters, USA). DMF was used as the eluent and PS was used as the standard. The flow rate was 1 mL/min. Differential scanning calorimetry (DSC) curves were performed on a TA DSC2500. The thermal stabilities of the samples were evaluated by TG with TGA/DSC1/110LF apparatus under nitrogen atmosphere and air atmosphere. LOI measurements were processed on the Oxygen Index Flammability Gauge (JF-6) according to ASTM D 2863-97 standard. The samples were molded with a size of 120 × 6.5 × 3.2 mm^3^. The vertical burning test (UL-94) was surveyed on a vertical burning instrument (KB-RS) according to ASTM D3801 standard. The size of samples was 120 × 12.7 × 3.2 mm^3^. The mechanical properties of PET and copolyester were determined using INSTRON F563-44 according to ASTM D-638 at 5 mm/min cross-head speed. The specimen has a width of 4 mm and thickness of 2 mm.

**Life Cycle Assessment (LCA)**

The LCA of Kapton recycling was assessed using the method of the Institute of Environmental Sciences (CML2001)[3], following the International Organization for Standards ISO14044[4]. The functional unit of 1 kg of waste Kapton is used in the scope of the LCA. The waste is assumed to be unburden, and the impact of gas emissions is overlooked. Considering that excess MEA can be recovered by distillation, MEA is calculated as net consumption. The life cycle inventory data for inputs and outputs are obtained from bench-scale experiments, literature, patents and database Ecoinvent 3.9[1, 5-7]. In the absence of specific data on the production of NNHPI, its data is estimated by tracing its preparation process. Major inputs and outputs for this ammonolysis-coupled hydrolysis recovery process are described in Table S8.

**Density functional theory (DFT) calculations**

All data were calculated with the Gaussian 16 software package and optimized at the B3LYP level of DFT[8-10]. The basis set 6-31G was selected for all non-metal atoms. Vibrational frequency analysis was computed to ensure the points that the minimum has no imaginary frequency. All the energetic values reported in this work are Gibbs free energies at 298.15 K.

**Optimized cartesian coordinates for hydrolysis of PETI**

**H^+^**

| 1 | 0.000000000 | 0.000000000 | 0.000000000 |
| --- | --- | --- | --- |

**H_2_O**

| 8 | 0.000000000 | 0.000000000 | 0.114322000 |
| --- | --- | --- | --- |
| 1 | 0.000000000 | 0.790917000 | -0.457290000 |
| 1 | 0.000000000 | -0.790917000 | -0.457290000 |

| 8 | 1.853614000 | -0.258554000 | -0.000024000 |
| --- | --- | --- | --- |
| 1 | 2.670583000 | 0.277639000 | 0.000131000 |
| 6 | 0.662941000 | 0.544953000 | 0.000003000 |
| 1 | 0.588767000 | 1.184152000 | 0.892863000 |
| 1 | 0.588750000 | 1.184170000 | -0.892859000 |
| 6 | -0.432441000 | -0.521374000 | 0.000012000 |
| 1 | -0.356137000 | -1.147409000 | 0.890440000 |
| 1 | -0.356199000 | -1.147282000 | -0.890505000 |
| 7 | -1.834407000 | 0.112940000 | 0.000000000 |
| 1 | -2.559623000 | -0.614796000 | 0.000719000 |
| 1 | -1.973352000 | 0.700500000 | 0.831679000 |
| 1 | -1.973855000 | 0.699404000 | -0.832366000 |

| 6 | 4.489804000 | -3.385769000 | 0.983945000 |
| --- | --- | --- | --- |
| 6 | 3.526394000 | -2.540850000 | 0.416627000 |
| 6 | 3.861936000 | -1.268006000 | -0.046359000 |
| 6 | 5.188531000 | -0.837614000 | 0.075462000 |
| 6 | 6.172235000 | -1.656191000 | 0.639881000 |
| 6 | 5.805370000 | -2.932788000 | 1.087208000 |
| 1 | 4.191742000 | -4.368473000 | 1.327385000 |
| 1 | 3.131623000 | -0.608488000 | -0.495595000 |
| 1 | 7.193322000 | -1.308669000 | 0.729155000 |
| 1 | 6.559538000 | -3.577774000 | 1.526705000 |
| 8 | 2.237989000 | -3.084120000 | 0.291427000 |
| 8 | 5.435786000 | 0.457471000 | -0.406439000 |
| 6 | 1.092138000 | -2.269045000 | 0.192951000 |
| 6 | 0.829126000 | -1.235572000 | 1.096399000 |
| 6 | 0.161978000 | -2.598603000 | -0.796202000 |
| 6 | -0.363091000 | -0.511395000 | 1.008630000 |
| 1 | 1.549943000 | -0.993853000 | 1.868946000 |
| 6 | -1.032676000 | -1.887185000 | -0.881027000 |
| 1 | 0.381724000 | -3.410706000 | -1.479001000 |
| 6 | -1.306334000 | -0.833164000 | 0.013990000 |
| 1 | -0.576931000 | 0.288546000 | 1.701248000 |
| 1 | -1.764232000 | -2.145813000 | -1.640556000 |
| 6 | 6.749872000 | 0.984090000 | -0.404240000 |
| 6 | 7.153423000 | 1.820686000 | 0.638371000 |
| 6 | 7.604118000 | 0.738725000 | -1.482110000 |
| 6 | 8.416449000 | 2.412784000 | 0.607051000 |
| 1 | 6.473324000 | 2.011561000 | 1.460868000 |
| 6 | 8.868504000 | 1.327185000 | -1.515588000 |
| 1 | 7.272466000 | 0.097998000 | -2.291542000 |
| 6 | 9.297063000 | 2.174282000 | -0.470291000 |
| 1 | 8.725566000 | 3.066792000 | 1.417296000 |
| 1 | 9.529361000 | 1.137374000 | -2.356466000 |
| 7 | 10.552407000 | 2.760013000 | -0.502741000 |
| 1 | 10.857058000 | 3.372632000 | 0.233017000 |
| 1 | 11.177733000 | 2.602142000 | -1.273124000 |
| 7 | -2.539244000 | -0.160643000 | -0.159016000 |
| 1 | -3.106150000 | -0.479437000 | -0.948204000 |
| 6 | -3.073743000 | 0.829409000 | 0.615107000 |
| 8 | -2.484325000 | 1.331718000 | 1.608012000 |
| 6 | -4.423680000 | 1.392190000 | 0.209505000 |
| 6 | -4.578819000 | 2.750422000 | 0.534809000 |
| 6 | -5.532232000 | 0.700250000 | -0.349780000 |
| 6 | -5.756792000 | 3.463917000 | 0.283546000 |
| 1 | -3.738709000 | 3.240635000 | 1.013206000 |
| 6 | -6.728180000 | 1.410865000 | -0.577127000 |
| 6 | -6.836852000 | 2.768472000 | -0.279545000 |
| 1 | -7.591760000 | 0.889376000 | -0.975718000 |
| 1 | -7.771396000 | 3.284801000 | -0.477512000 |
| 6 | -5.851546000 | 4.938451000 | 0.600291000 |
| 1 | -5.507940000 | 5.546125000 | -0.247799000 |
| 1 | -6.882532000 | 5.235868000 | 0.818340000 |
| 1 | -5.229621000 | 5.201931000 | 1.461725000 |
| 6 | -5.488819000 | -0.732118000 | -0.788788000 |
| 7 | -6.444260000 | -1.610957000 | -0.351163000 |
| 1 | -6.300022000 | -2.548649000 | -0.713112000 |
| 8 | -4.610995000 | -1.155014000 | -1.596350000 |
| 6 | -7.466586000 | -1.445557000 | 0.689631000 |
| 1 | -7.257249000 | -2.128802000 | 1.524260000 |
| 1 | -7.410207000 | -0.426604000 | 1.071576000 |
| 6 | -8.871079000 | -1.729452000 | 0.142975000 |
| 1 | -8.963639000 | -2.789055000 | -0.139038000 |
| 1 | -9.617035000 | -1.518511000 | 0.912105000 |
| 8 | -9.214627000 | -0.871904000 | -0.979069000 |
| 1 | -8.657989000 | -1.105552000 | -1.750303000 |

| 6 | 1.489796000 | -1.647104000 | -0.358721000 |
| --- | --- | --- | --- |
| 6 | 2.012903000 | -0.773831000 | 0.605136000 |
| 6 | 3.395593000 | -0.683698000 | 0.815109000 |
| 6 | 4.233522000 | -1.475501000 | 0.034734000 |
| 6 | 3.750570000 | -2.358531000 | -0.930264000 |
| 6 | 2.364715000 | -2.435529000 | -1.113670000 |
| 1 | 0.420052000 | -1.710959000 | -0.511615000 |
| 1 | 3.777409000 | -0.027516000 | 1.586905000 |
| 1 | 4.437201000 | -2.972550000 | -1.499212000 |
| 1 | 1.961481000 | -3.116315000 | -1.855085000 |
| 8 | 1.237826000 | 0.077513000 | 1.387989000 |
| 8 | 5.641390000 | -1.462688000 | 0.279694000 |
| 6 | -0.163093000 | -0.114116000 | 1.520067000 |
| 6 | -1.008388000 | 0.890938000 | 1.050050000 |
| 6 | -0.666343000 | -1.226932000 | 2.199051000 |
| 6 | -2.385246000 | 0.789639000 | 1.263560000 |
| 1 | -0.583519000 | 1.758818000 | 0.559644000 |
| 6 | -2.045683000 | -1.341578000 | 2.383270000 |
| 1 | 0.007809000 | -1.986084000 | 2.579045000 |
| 6 | -2.911886000 | -0.338297000 | 1.915528000 |
| 1 | -3.051160000 | 1.592566000 | 0.969884000 |
| 1 | -2.455815000 | -2.207161000 | 2.892946000 |
| 6 | 6.421453000 | -0.374817000 | -0.030229000 |
| 6 | 7.759883000 | -0.451924000 | 0.400921000 |
| 6 | 5.964812000 | 0.744400000 | -0.745850000 |
| 6 | 8.641895000 | 0.586536000 | 0.124086000 |
| 1 | 8.079622000 | -1.330013000 | 0.947236000 |
| 6 | 6.848316000 | 1.789002000 | -1.024882000 |
| 1 | 4.936619000 | 0.799830000 | -1.077555000 |
| 6 | 8.166775000 | 1.692917000 | -0.586169000 |
| 1 | 9.672473000 | 0.523478000 | 0.460977000 |
| 1 | 6.493838000 | 2.654184000 | -1.578861000 |
| 7 | 9.114997000 | 2.826126000 | -0.877155000 |
| 1 | 9.955253000 | 2.495424000 | -1.369718000 |
| 1 | 8.657626000 | 3.526483000 | -1.473788000 |
| 7 | -4.306618000 | -0.454271000 | 2.167058000 |
| 1 | -4.596532000 | -0.650180000 | 3.118887000 |
| 6 | -5.360871000 | -0.114404000 | 1.338344000 |
| 8 | -6.511970000 | 0.020186000 | 1.797153000 |
| 6 | -5.125278000 | -0.087486000 | -0.150529000 |
| 6 | -4.804199000 | -1.291960000 | -0.787791000 |
| 6 | -5.433433000 | 1.044643000 | -0.934836000 |
| 6 | -4.802071000 | -1.408582000 | -2.188206000 |
| 1 | -4.581848000 | -2.164713000 | -0.181523000 |
| 6 | -5.445098000 | 0.934081000 | -2.333851000 |
| 6 | -5.131800000 | -0.276426000 | -2.951591000 |
| 1 | -5.689746000 | 1.806786000 | -2.926822000 |
| 1 | -5.142724000 | -0.344520000 | -4.035006000 |
| 6 | -4.496761000 | -2.731642000 | -2.851322000 |
| 1 | -5.418297000 | -3.302552000 | -3.027241000 |
| 1 | -4.012458000 | -2.590565000 | -3.823136000 |
| 1 | -3.844009000 | -3.352446000 | -2.229229000 |
| 6 | -5.640953000 | 2.353897000 | -0.287633000 |
| 8 | -5.175857000 | 2.686478000 | 0.814412000 |
| 1 | 9.418339000 | 3.298498000 | -0.014694000 |
| 8 | -6.368022000 | 3.234017000 | -1.054619000 |
| 1 | -6.461980000 | 4.090291000 | -0.584259000 |

| 6 | 1.672083000 | -1.915866000 | 0.269843000 |
| --- | --- | --- | --- |
| 8 | 2.779508000 | -2.445309000 | 0.118093000 |
| 6 | 1.367464000 | -0.485650000 | 0.026379000 |
| 6 | 2.429948000 | 0.414871000 | 0.183081000 |
| 6 | 0.079971000 | -0.011414000 | -0.309894000 |
| 6 | 2.248683000 | 1.797580000 | 0.041649000 |
| 1 | 3.407154000 | 0.013059000 | 0.429413000 |
| 6 | -0.108280000 | 1.374636000 | -0.430446000 |
| 6 | 0.956951000 | 2.262195000 | -0.250655000 |
| 1 | -1.094246000 | 1.765382000 | -0.658162000 |
| 1 | 0.780309000 | 3.329318000 | -0.348968000 |
| 6 | 3.411357000 | 2.752673000 | 0.185366000 |
| 1 | 3.902932000 | 2.922225000 | -0.782068000 |
| 1 | 3.084291000 | 3.728083000 | 0.560320000 |
| 1 | 4.169645000 | 2.360698000 | 0.870718000 |
| 6 | -1.004792000 | -0.962883000 | -0.735754000 |
| 7 | -2.247971000 | -0.871888000 | -0.146888000 |
| 1 | -2.895915000 | -1.570300000 | -0.495757000 |
| 8 | -0.803857000 | -1.781851000 | -1.659823000 |
| 6 | -2.634746000 | -0.133897000 | 1.059197000 |
| 1 | -2.919085000 | -0.832994000 | 1.858076000 |
| 1 | -1.768635000 | 0.429795000 | 1.407693000 |
| 6 | -3.801358000 | 0.820102000 | 0.772313000 |
| 1 | -4.714939000 | 0.247973000 | 0.550094000 |
| 1 | -3.998744000 | 1.449415000 | 1.642760000 |
| 8 | -3.501361000 | 1.736824000 | -0.315322000 |
| 1 | -3.350456000 | 1.215105000 | -1.131343000 |
| 8 | 0.600428000 | -2.617319000 | 0.781787000 |
| 1 | 0.832060000 | -3.567389000 | 0.861090000 |

| 6 | -0.321364000 | -0.785051000 | -0.013485000 |
| --- | --- | --- | --- |
| 6 | 0.658754000 | -1.786905000 | -0.076269000 |
| 6 | 2.015536000 | -1.463496000 | -0.073983000 |
| 6 | 2.436447000 | -0.125405000 | 0.001371000 |
| 6 | 1.453152000 | 0.874924000 | 0.029991000 |
| 6 | 0.086387000 | 0.566382000 | -0.000779000 |
| 1 | 0.337296000 | -2.821656000 | -0.108047000 |
| 1 | 2.755125000 | -2.256913000 | -0.120981000 |
| 1 | 1.741728000 | 1.920341000 | 0.054396000 |
| 6 | 3.903869000 | 0.229372000 | 0.060481000 |
| 1 | 4.262641000 | 0.241859000 | 1.098530000 |
| 1 | 4.513351000 | -0.497329000 | -0.486458000 |
| 1 | 4.094060000 | 1.221142000 | -0.361444000 |
| 6 | -0.843917000 | 1.720256000 | -0.096635000 |
| 6 | -1.726388000 | -1.240095000 | 0.109440000 |
| 8 | -0.679140000 | 2.813652000 | 0.455969000 |
| 8 | -2.184099000 | -2.276456000 | -0.386828000 |
| 8 | -1.882131000 | 1.497348000 | -0.973739000 |
| 1 | -2.474719000 | 2.279668000 | -0.994002000 |
| 8 | -2.480043000 | -0.440719000 | 0.941601000 |
| 1 | -3.401026000 | -0.777607000 | 0.981198000 |

| 6 | 5.399707000 | -2.411192000 | -0.372784000 |
| --- | --- | --- | --- |
| 6 | 4.142519000 | -1.935517000 | 0.007720000 |
| 6 | 3.935595000 | -0.584158000 | 0.290565000 |
| 6 | 4.999901000 | 0.303511000 | 0.128080000 |
| 6 | 6.268118000 | -0.129970000 | -0.251625000 |
| 6 | 6.456032000 | -1.499736000 | -0.486033000 |
| 1 | 5.533067000 | -3.466153000 | -0.575841000 |
| 1 | 2.969516000 | -0.237654000 | 0.627411000 |
| 1 | 7.074500000 | 0.583975000 | -0.360246000 |
| 1 | 7.436431000 | -1.857477000 | -0.779451000 |
| 8 | 3.094160000 | -2.870818000 | 0.130971000 |
| 8 | 4.821067000 | 1.690624000 | 0.395010000 |
| 6 | 1.768412000 | -2.403918000 | 0.064500000 |
| 6 | 0.931640000 | -2.620422000 | 1.161051000 |
| 6 | 1.297202000 | -1.756494000 | -1.085233000 |
| 6 | -0.380331000 | -2.138906000 | 1.130023000 |
| 1 | 1.315653000 | -3.145747000 | 2.026659000 |
| 6 | -0.012963000 | -1.280099000 | -1.115804000 |
| 1 | 1.950319000 | -1.635481000 | -1.941150000 |
| 6 | -0.841838000 | -1.448140000 | 0.002149000 |
| 1 | -1.035811000 | -2.293272000 | 1.978388000 |
| 1 | -0.394804000 | -0.786989000 | -2.002162000 |
| 6 | 3.550618000 | 2.235892000 | 0.225348000 |
| 6 | 3.015992000 | 2.992106000 | 1.273323000 |
| 6 | 2.825223000 | 2.037662000 | -0.960972000 |
| 6 | 1.717386000 | 3.500092000 | 1.164544000 |
| 1 | 3.605593000 | 3.147553000 | 2.167852000 |
| 6 | 1.526981000 | 2.530453000 | -1.067002000 |
| 1 | 3.265367000 | 1.474669000 | -1.773978000 |
| 6 | 0.985436000 | 3.237251000 | 0.008130000 |
| 1 | 1.289214000 | 4.063027000 | 1.988926000 |
| 1 | 0.944633000 | 2.342016000 | -1.962778000 |
| 7 | -0.445520000 | 3.652894000 | -0.075230000 |
| 1 | -0.726296000 | 4.156217000 | 0.771888000 |
| 1 | -0.615775000 | 4.267813000 | -0.878902000 |
| 7 | -2.177924000 | -0.924481000 | -0.018021000 |
| 6 | -3.360154000 | -1.742640000 | 0.145722000 |
| 8 | -3.343419000 | -2.955771000 | 0.336389000 |
| 6 | -4.504406000 | -0.806267000 | 0.043870000 |
| 6 | -5.863616000 | -1.055258000 | 0.118126000 |
| 6 | -3.998401000 | 0.489886000 | -0.159328000 |
| 6 | -6.759600000 | 0.033306000 | -0.017128000 |
| 1 | -6.234786000 | -2.062684000 | 0.275084000 |
| 6 | -4.858379000 | 1.572228000 | -0.294525000 |
| 6 | -6.239478000 | 1.326620000 | -0.220718000 |
| 1 | -4.485542000 | 2.577488000 | -0.457522000 |
| 1 | -6.930966000 | 2.155959000 | -0.325485000 |
| 6 | -8.248687000 | -0.201342000 | 0.053406000 |
| 1 | -8.809097000 | 0.732594000 | -0.035815000 |
| 1 | -8.579825000 | -0.870932000 | -0.749763000 |
| 1 | -8.529287000 | -0.675064000 | 1.001659000 |
| 6 | -2.524313000 | 0.412613000 | -0.187368000 |
| 8 | -1.695505000 | 1.348650000 | -0.331953000 |
| 1 | -1.060009000 | 2.784391000 | -0.173555000 |

| 7 | -1.304368000 | -0.004756000 | 0.358150000 |
| --- | --- | --- | --- |
| 6 | -0.413014000 | 1.087193000 | 0.295694000 |
| 8 | -0.740455000 | 2.279370000 | 0.379486000 |
| 6 | 0.941351000 | 0.493924000 | 0.116630000 |
| 6 | 2.176736000 | 1.109511000 | -0.002256000 |
| 6 | 0.807974000 | -0.903442000 | 0.079005000 |
| 6 | 3.322897000 | 0.298212000 | -0.164769000 |
| 1 | 2.260900000 | 2.191234000 | 0.029536000 |
| 6 | 1.917756000 | -1.720477000 | -0.079935000 |
| 6 | 3.173484000 | -1.101606000 | -0.201329000 |
| 1 | 1.818164000 | -2.799894000 | -0.108979000 |
| 1 | 4.058329000 | -1.717878000 | -0.327105000 |
| 6 | 4.688599000 | 0.934080000 | -0.292928000 |
| 1 | 5.466163000 | 0.183242000 | -0.459743000 |
| 1 | 4.720015000 | 1.643191000 | -1.129058000 |
| 1 | 4.951816000 | 1.493542000 | 0.613526000 |
| 6 | -0.631105000 | -1.241420000 | 0.232860000 |
| 8 | -1.179894000 | -2.352148000 | 0.254949000 |
| 6 | -2.745342000 | 0.124922000 | 0.555167000 |
| 1 | -2.931657000 | 1.027010000 | 1.142195000 |
| 1 | -3.096244000 | -0.746815000 | 1.111999000 |
| 6 | -3.503479000 | 0.217478000 | -0.769348000 |
| 1 | -3.325413000 | -0.687371000 | -1.366430000 |
| 1 | -3.158371000 | 1.091985000 | -1.337923000 |
| 8 | -4.902249000 | 0.342959000 | -0.396578000 |
| 1 | -5.467485000 | 0.403341000 | -1.190670000 |

| 6 | 1.122101000 | 1.718861000 | 0.471593000 |
| --- | --- | --- | --- |
| 6 | 1.107022000 | 0.321040000 | 0.451664000 |
| 6 | 0.000015000 | -0.398203000 | 0.000076000 |
| 6 | -1.107005000 | 0.320932000 | -0.451664000 |
| 6 | -1.122083000 | 1.718748000 | -0.471904000 |
| 6 | 0.000013000 | 2.410129000 | -0.000246000 |
| 1 | 1.980135000 | 2.255032000 | 0.859298000 |
| 1 | 0.000017000 | -1.480150000 | 0.000204000 |
| 1 | -1.980117000 | 2.254838000 | -0.859721000 |
| 1 | 0.000008000 | 3.494023000 | -0.000371000 |
| 8 | 2.174743000 | -0.433079000 | 0.996673000 |
| 8 | -2.174717000 | -0.433321000 | -0.996488000 |
| 6 | 3.476417000 | -0.347986000 | 0.533055000 |
| 6 | 4.441769000 | -0.945344000 | 1.362223000 |
| 6 | 3.841127000 | 0.238111000 | -0.687477000 |
| 6 | 5.778280000 | -0.956856000 | 0.975204000 |
| 1 | 4.125231000 | -1.392478000 | 2.295812000 |
| 6 | 5.183571000 | 0.229517000 | -1.078240000 |
| 1 | 3.096389000 | 0.689134000 | -1.330117000 |
| 6 | 6.125572000 | -0.365507000 | -0.242732000 |
| 1 | 6.521006000 | -1.418563000 | 1.618797000 |
| 1 | 5.465491000 | 0.679790000 | -2.025875000 |
| 6 | -3.476410000 | -0.348106000 | -0.532954000 |
| 6 | -4.441731000 | -0.945654000 | -1.362017000 |
| 6 | -3.841164000 | 0.238267000 | 0.687435000 |
| 6 | -5.778260000 | -0.957077000 | -0.975041000 |
| 1 | -4.125161000 | -1.393023000 | -2.295482000 |
| 6 | -5.183620000 | 0.229771000 | 1.078150000 |
| 1 | -3.096441000 | 0.689414000 | 1.330006000 |
| 6 | -6.125590000 | -0.365460000 | 0.242748000 |
| 1 | -6.520958000 | -1.418955000 | -1.618544000 |
| 1 | -5.465573000 | 0.680238000 | 2.025682000 |
| 7 | -7.571722000 | -0.373717000 | 0.661407000 |
| 1 | -7.944939000 | -1.331852000 | 0.707004000 |
| 1 | -7.679019000 | 0.046053000 | 1.593531000 |
| 1 | -8.159181000 | 0.163032000 | 0.008238000 |
| 7 | 7.571685000 | -0.373872000 | -0.661444000 |
| 1 | 8.159180000 | 0.162991000 | -0.008402000 |
| 1 | 7.944883000 | -1.332023000 | -0.706869000 |
| 1 | 7.678957000 | 0.045716000 | -1.593653000 |

**Supporting Figures and Tables**


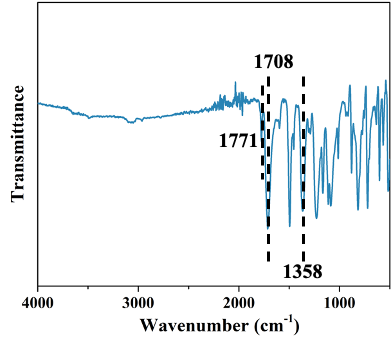


**Figure S1** The FTIR spectrum of Kapton film.

The chemical structures of Kapton film were characterized in reflection modes using FT-IR. The peaks of C=O symmetric and asymmetric stretching vibrations are observed at around 1771 cm^-1^ and 1708 cm^-1^, respectively. The peak at around 1353 cm^-1^ is attributed to the C-N stretching vibration. The appearances of these peaks indicate the presence of imide ring in Kapton film.





**Figure S2** The TGA curve of Kapton film.

2 g Kapton film and 20 mL ethylenediamine were added into a round-bottom flask, and the reaction was performed at 110 ℃ for 3 h. The aminolysis products were concentrated by distillation under reduced pressure, and then characterized by NMR.

**
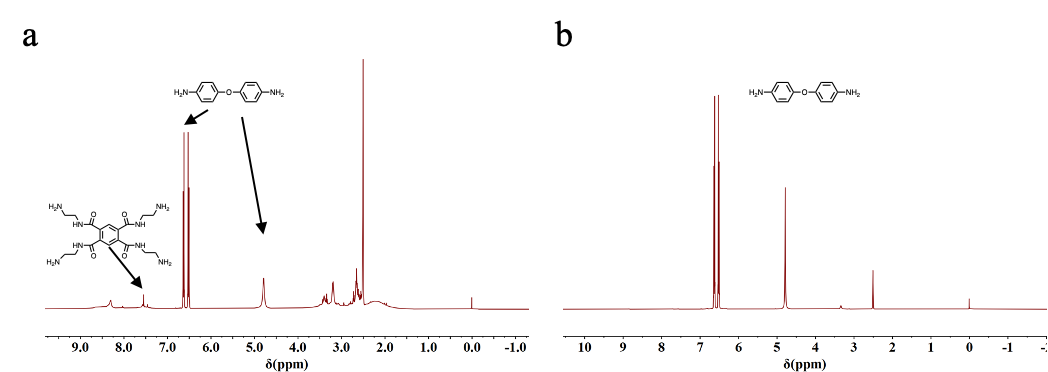
**

**Figure S3** ^1^H-NMR spectrum of aminolysis products obtained from the degradation of Kapton film by ethylenediamine.

2 g Kapton film and 20 mL 1,3-diaminopropane were added into a round-bottom flask, and the reaction was performed at 120 ℃ for 3 h. The aminolysis products were concentrated by distillation under reduced pressure, and then characterized by NMR.

**
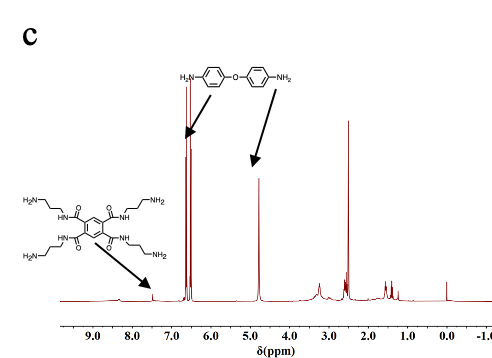
**

**Figure S4** ^1^H-NMR spectrum of aminolysis products obtained from the degradation of Kapton film by 1,3-diaminopropane.

2 g Kapton film and 15 mL 2 M HCl were added into a round-bottom flask equipped with a magnetic stir bar. Subsequently, the reaction was performed at 100 ℃ for 3 h.


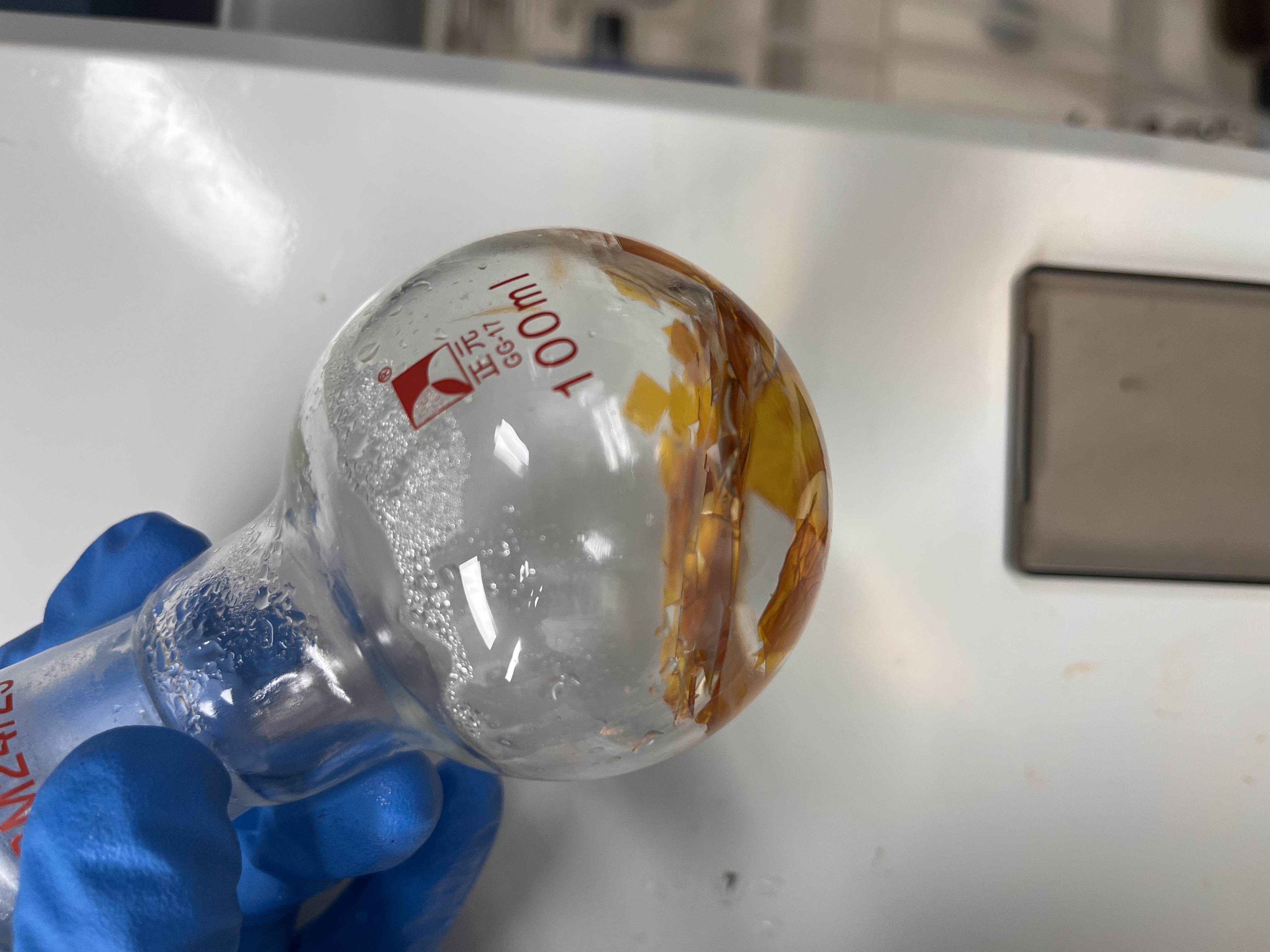


**Figure S5** Photograph of Kapton film directly hydrolyzed in HCl at 100℃ for 3 h.

2 g Kapton film and 20 mL MEA were added into a round-bottom flask, and the reaction was performed at 120 ℃ for 3 h. The aminolysis products were concentrated by distillation under reduced pressure, and then characterized by NMR.


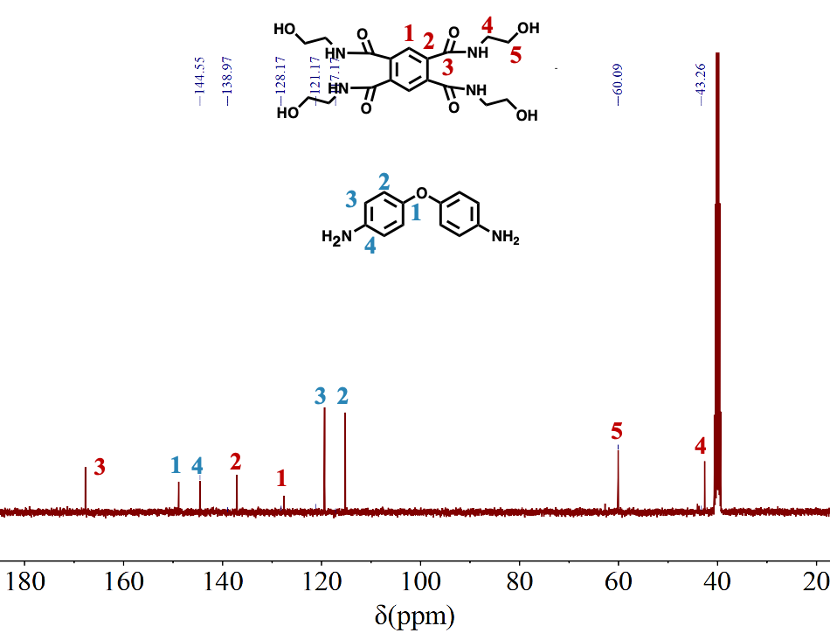


**Figure S6** The ^13^C-NMR spectrum of aminolysis products obtained from the degradation of Kapton film by MEA.

2 g Kapton film and 20 mL MEA were added into a round-bottom flask, and the reaction was performed at 120 ℃ for 3 h. The aminolysis products was concentrated by distillation under reduced pressure. Then, 15 mL 2M HCl was added and reacted with the aminolysis products at 100 ℃ for 1 h. After that, NNHPI was collected by filtration. The 4,4’-ODA was precipitated from the filtrate after adding NaOH to alkaline and characterized by GC-MS.

**
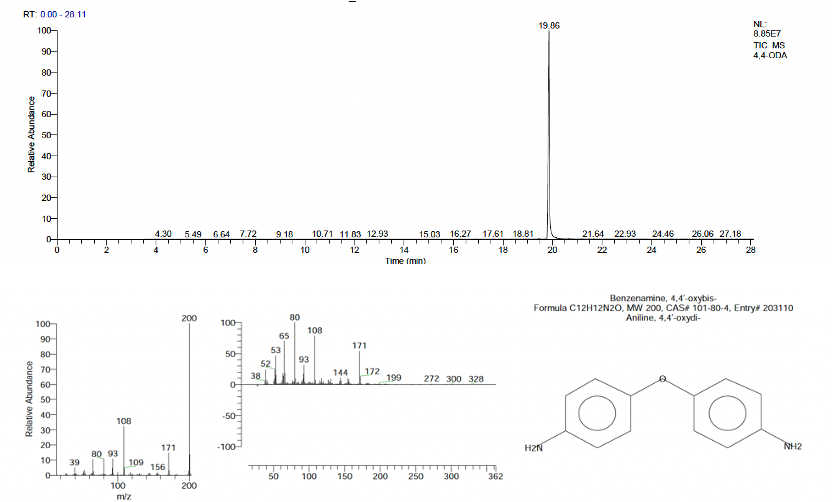
**

**Figure S7** The GC-MS spectrum of recycled 4,4’-ODA.

2 g Kapton film and 20 mL MEA were added into a round-bottom flask, and the reaction was performed at 120 ℃ for 3 h. The aminolysis products was concentrated by distillation under reduced pressure. Then, 15 mL 2M HCl was added and reacted with the aminolysis products at 100 ℃ for 1 h. After that, NNHPI was collected by filtration. The 4,4’-ODA was precipitated from the filtrate after adding NaOH to alkaline and characterized by NMR.


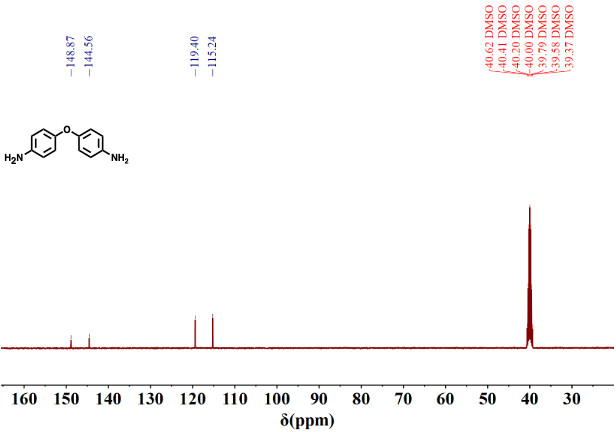


**Figure S8** The ^13^C-NMR spectrum of recycled 4,4’-ODA.

2 g Kapton film and 20 mL MEA were added into a round-bottom flask, and the reaction was performed at 120 ℃ for 3 h. The aminolysis products were concentrated by distillation under reduced pressure. Then, 15 mL HCl was added and reacted with the aminolysis products at 100 ℃ for 1 h. After that, NNHPI was collected by filtration and characterized by LC-MS.


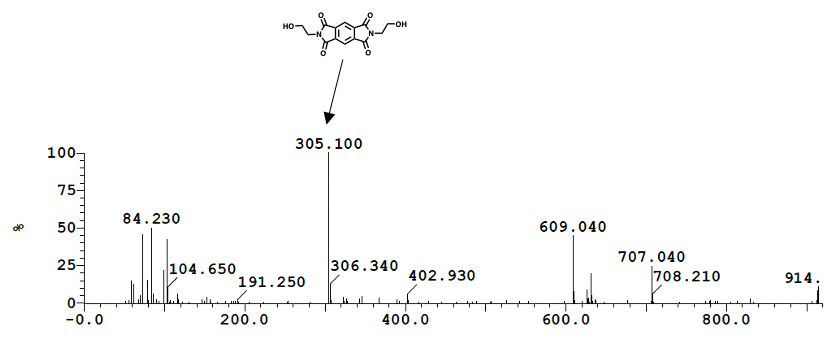


**Figure S9** The LC-MS spectrum of recycled NNHPI.

THPA was heated under 100 ℃ (simulated reaction temperature) and 170 ℃ (boiling point of MEA) for 1 h to observe its structure changes.

**
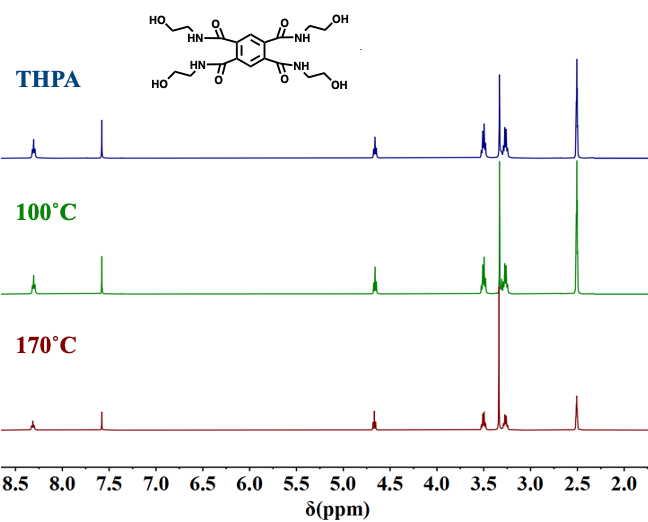
**

**Figure S10** The ^1^H-NMR spectra of THPA, THPA heated under 100 ℃ and 170 ℃ for 1 h, respectively.

2 g Kapton film and 20 mL MEA were added into a round-bottom flask, and the reaction was performed at 120 ℃ for 3 h. The aminolysis products were concentrated by distillation under reduced pressure. Then, 15 mL H_2_O was added and reacted with the aminolysis products at 100 ℃ for 1 h. After that, the filter cake was collected by filtration and characterized by NMR.

**
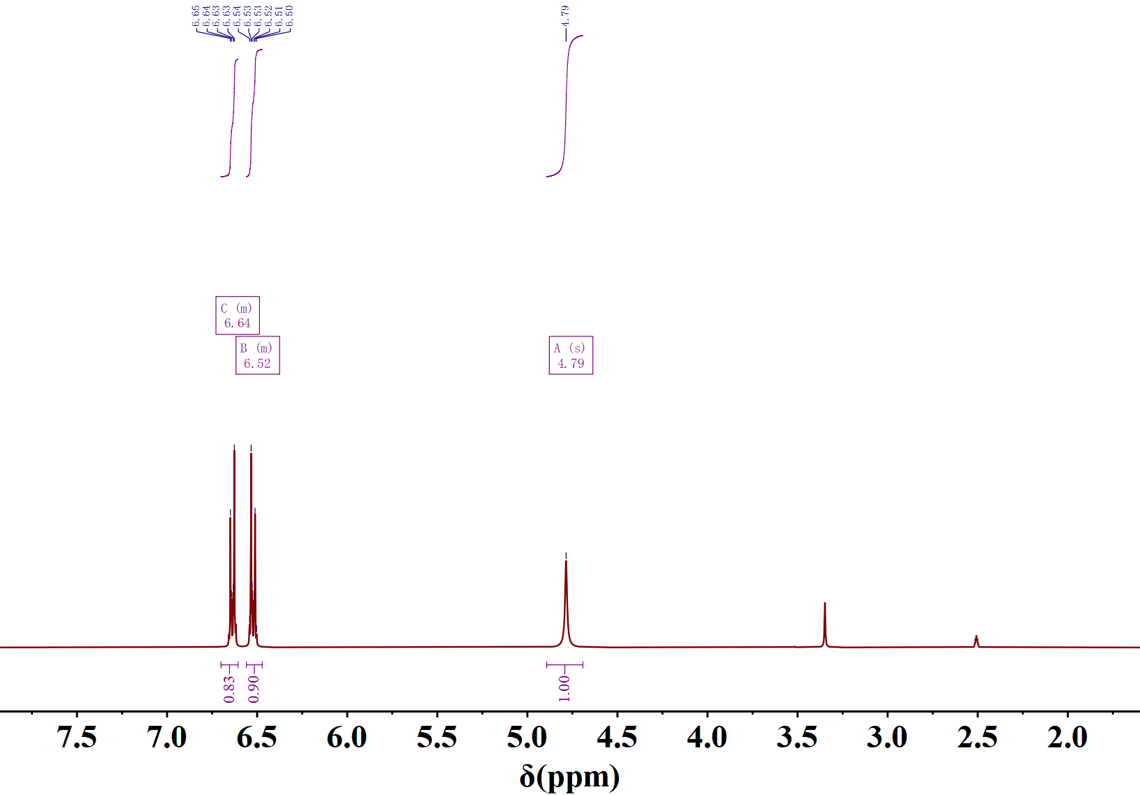
**

**Figure S11** The ^1^H-NMR spectrum of recycled 4,4’-ODA under neutral condition.

2 g Kapton film and 20 mL MEA were added into a round-bottom flask, and the reaction was performed at 120 ℃ for 3 h. The aminolysis products were concentrated by distillation under reduced pressure. Then, 15 mL 2 mol/L NaOH was added and reacted with the aminolysis products at 100 ℃ for 1 h. After that, the filter cake was collected by filtration and characterized by NMR.

**
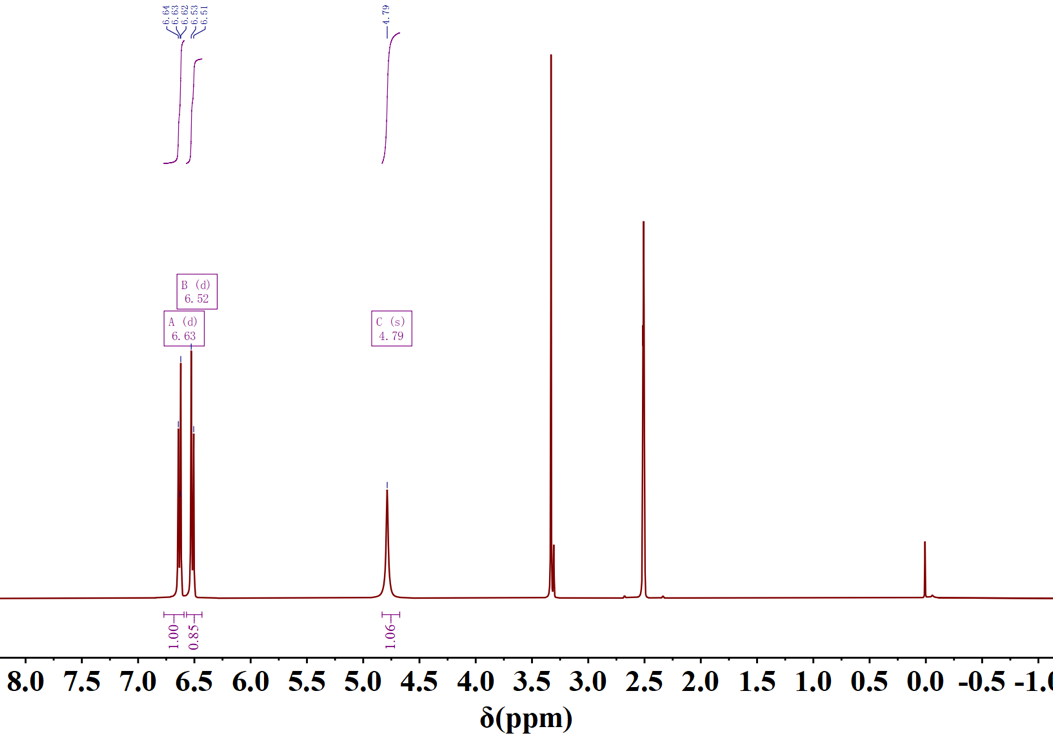
**

**Figure S12** The ^1^H-NMR spectrum of recycled 4,4’-ODA under alkaline condition.


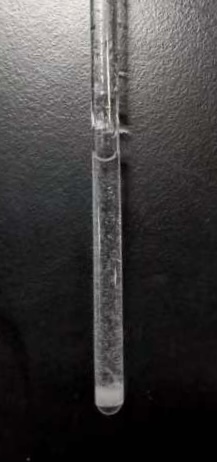


**Figure S13** The photograph of NNHPI precipitated from DCl.

2 g PETI and 20 mL MEA were added into a round-bottom flask, and the reaction was performed at 120 ℃ for 6 h. The resulting mixture was concentrated by distillation under reduced pressure. Then, 15 mL HCl was added and reacted with the mixture at 100 ℃ for 1 h. After that, DPETI was collected by filtration. The diamines were precipitated from the filtrate after adding NaOH to alkaline and characterized by NMR.

**
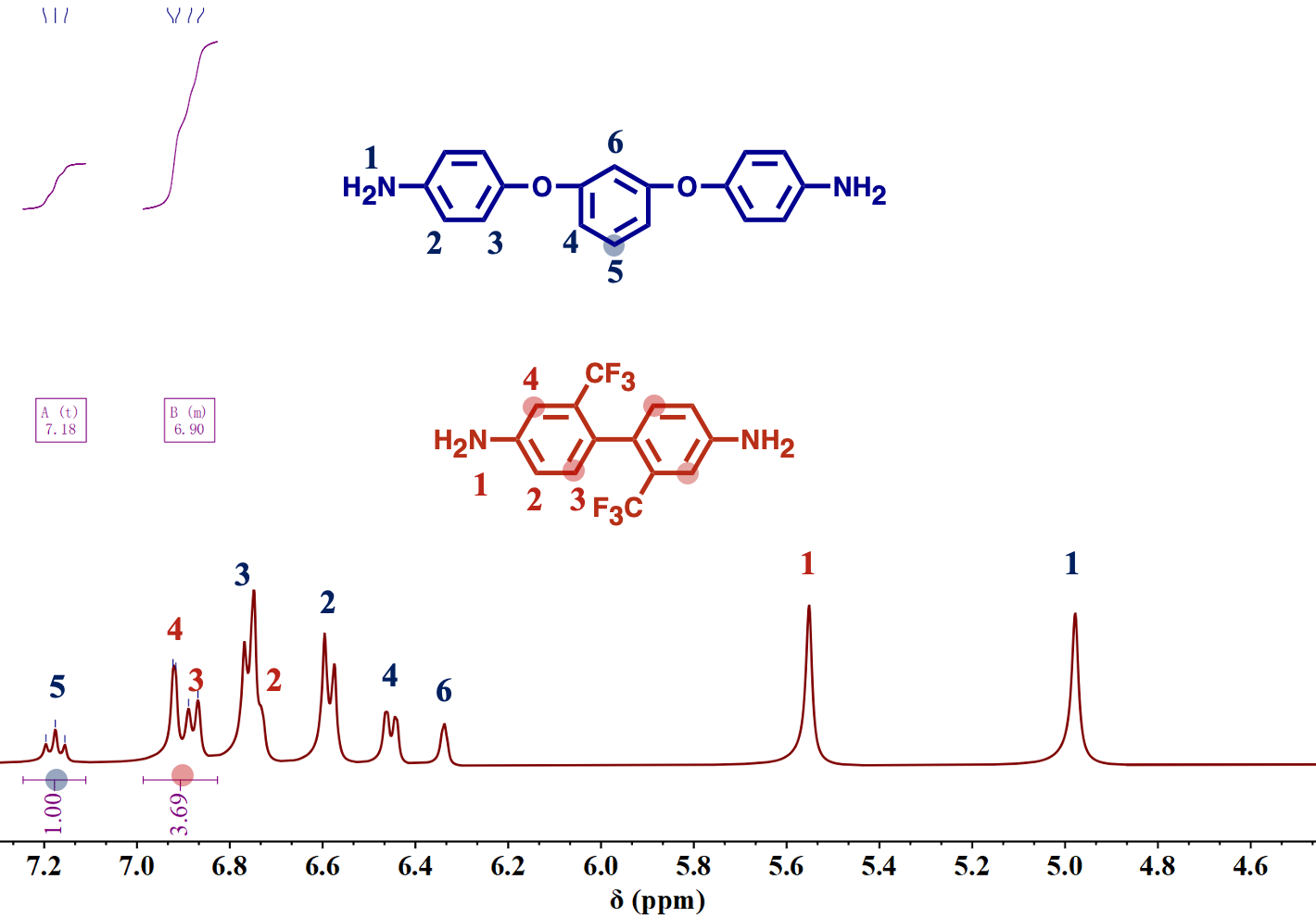
**

**Figure S14** The ^1^H-NMR spectrum of the recovered mixture of TFMB and 1,3,4-APB.

According to the results of nuclear magnetic integration, the radio of TFMB to 1,3,4-APB is 1:1.

2 g PETI and 20 mL MEA were added into a round-bottom flask, and the reaction was performed at 120 ℃ for 6 h. The resulting mixture was concentrated by distillation under reduced pressure. Then, 15 mL 2M HCl was added and reacted with the mixture at 100 ℃ for 1 h. After that, DPETI was collected by filtration. The diamines were precipitated from the filtrate after adding NaOH to alkaline. Then, the diamines were purified by column chromatography (0-75% EtOAc/Petroleum ether) to afford the TFMB.

**
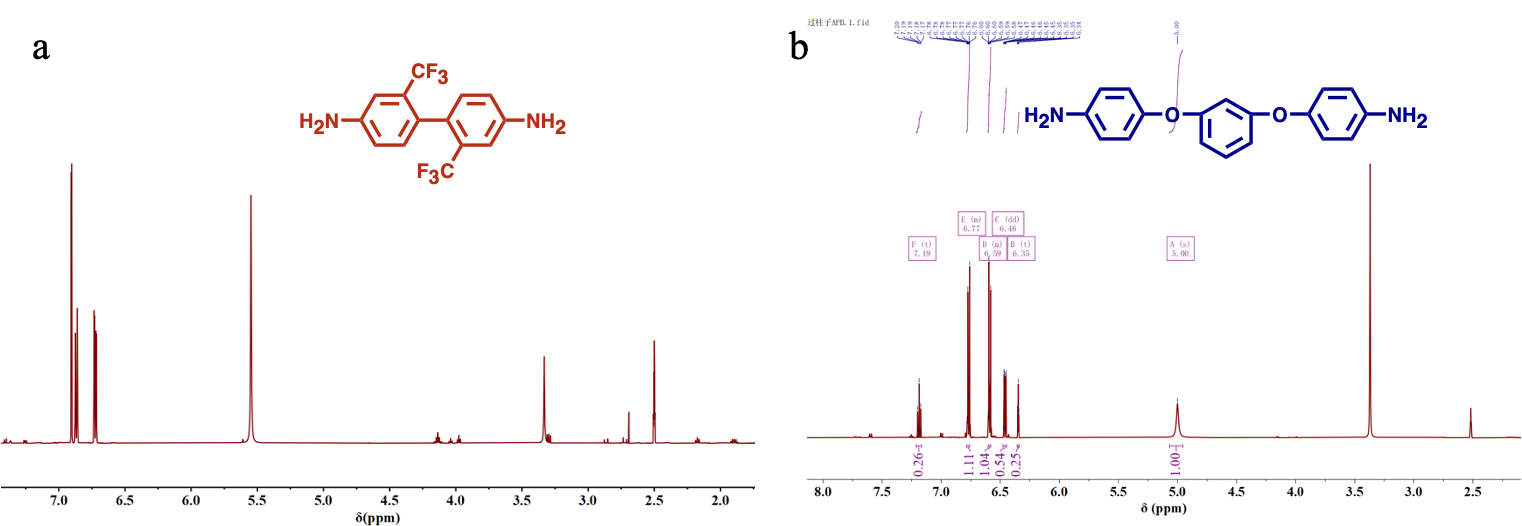
**

**Figure S15** ^1^H-NMR spectrum of recovered TFMB.

2 g PETI and 20 mL MEA were added into a round-bottom flask, and the reaction was performed at 120 ℃ for 6 h. The resulting mixture was concentrated by distillation under reduced pressure. Then, 15 mL 2M HCl was added and reacted with the mixture at 100 ℃ for 1 h. After that, DPETI was collected by filtration. The diamines were precipitated from the filtrate after adding NaOH to alkaline. Then, the diamines were purified by column chromatography (0-75% EtOAc/Petroleum ether) to afford the 1, 3, 4-APB.

**
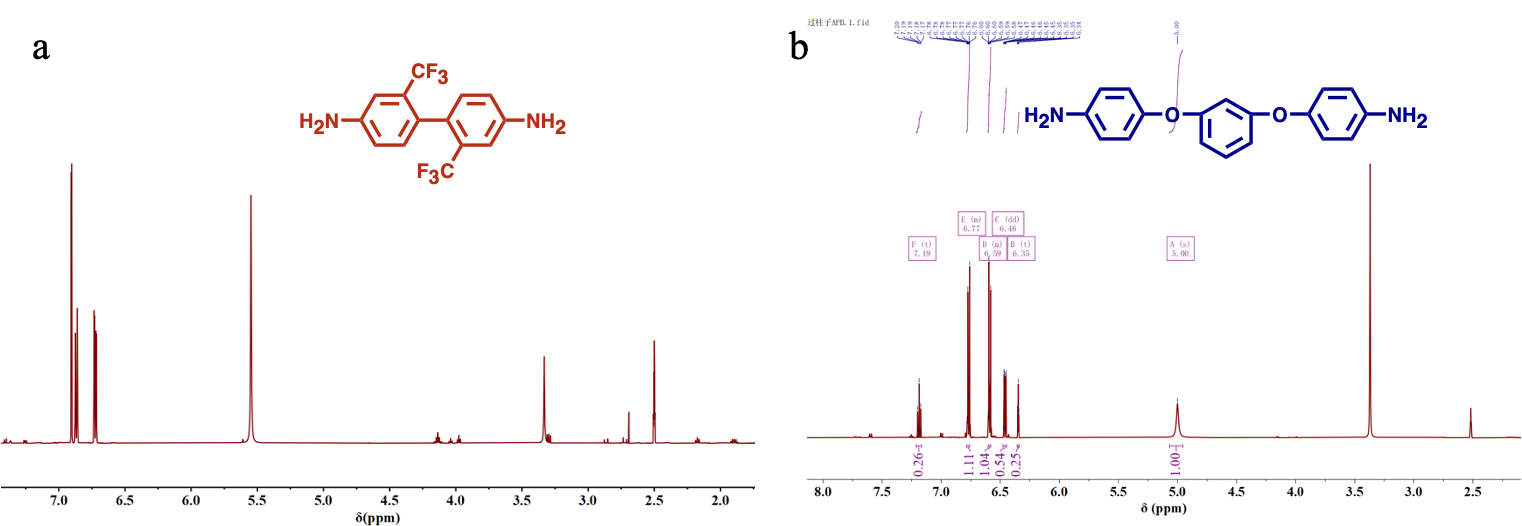
**

**Figure S16** ^1^H-NMR spectrum of recovered 1,3,4-APB.

**
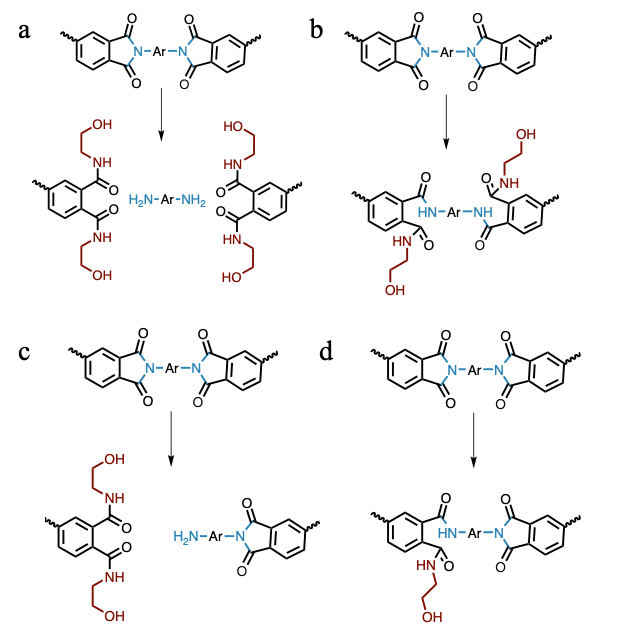
**

**Figure S17** Different modes of the breakage of imide ring.


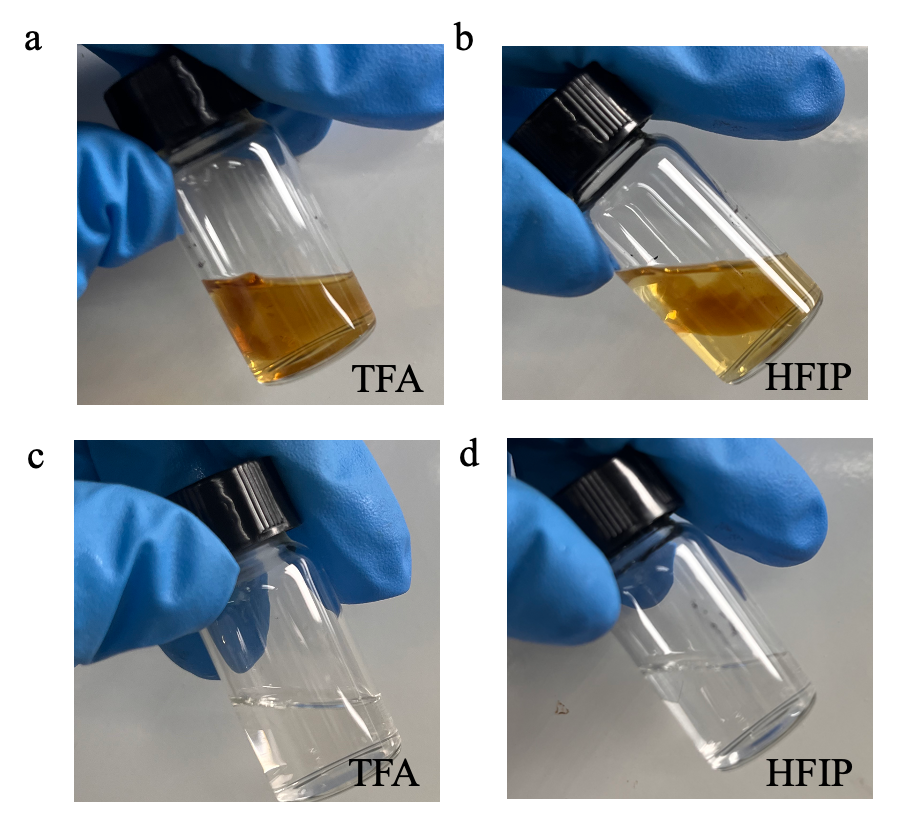


**Figure S18** The solubility of **a**, **b**, copolyesters and **c**, **d**, PET, in trifluoroacetic acid (TFA) and hexafluoroisopropanol (HFIP).

The PET is completely dissolved in TFA and HFIP, whereas copolyesters are not, indicating that the copolyesters contain cross-linked structures.


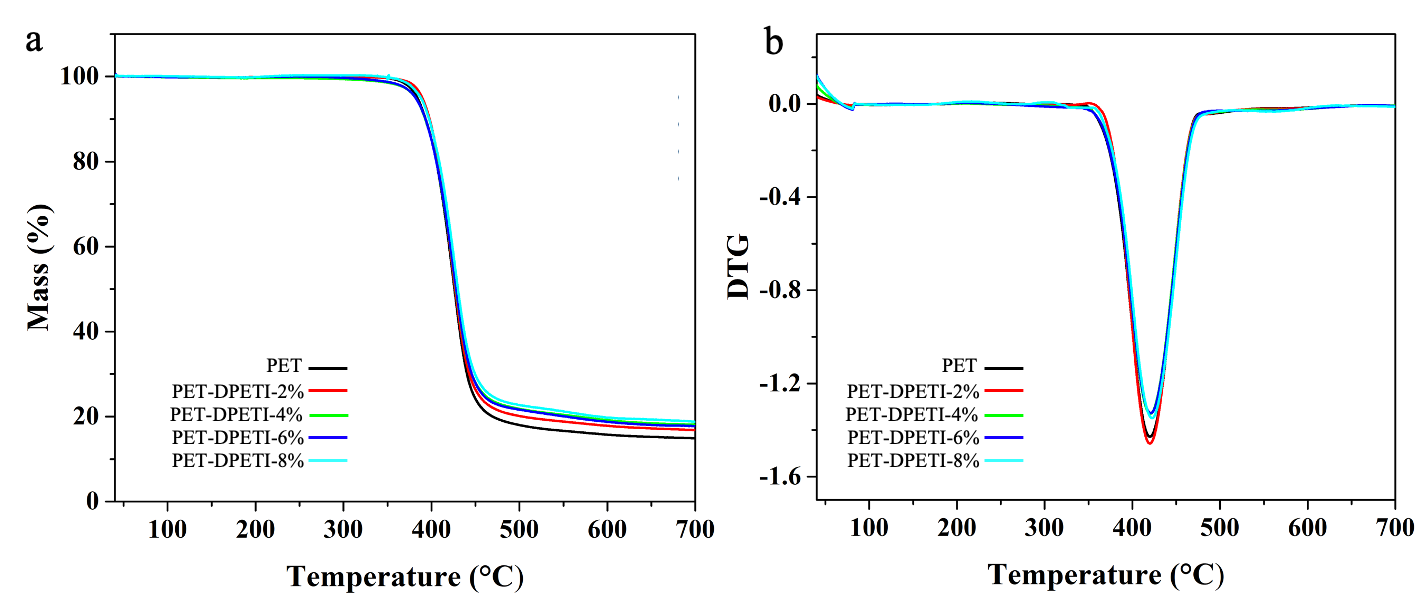
**Figure S19** **a**, TGA and **b**, DTG curves of PET-DPETI-n% in N_2_ atmosphere.


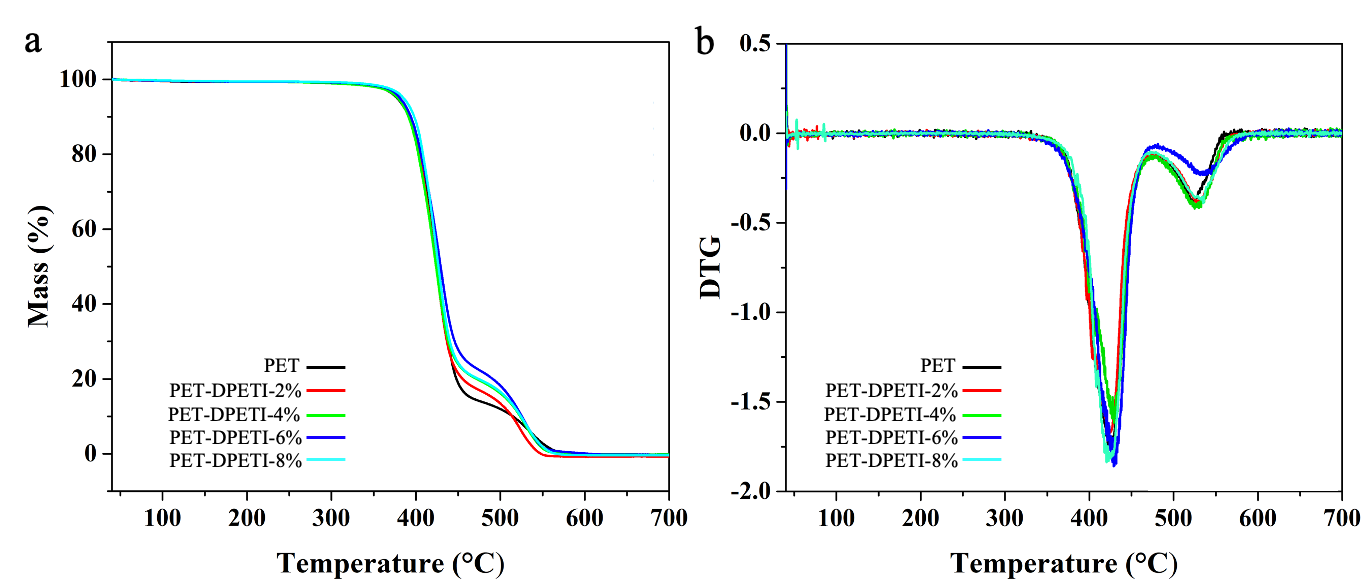


**Figure S20** **a**, TGA and **b**, DTG curves of PET-DPETI-n% in air atmosphere.

**Table S1** Comparison of degradation rate of Kapton film under different amines ^a^.

| **Entry** | **Amines** | **Boiling**  **point**  **(**℃**)** | **Aminolysis**  **temperature**  **(**℃**)** | **Aminolysis**  **time**  **(h)** | **Degradation**  **Rate ^b^**  **(%)** |
| --- | --- | --- | --- | --- | --- |
| 1 | Diethylamine | 55 | 50 | 8 | 0 |
| 2 | N-butylamine | 78 | 70 | 8 | 3 |
| 3 | Ethylenediamine | 116 | 110 | 3 | 100 |
| 4 | 1,3-diaminopropane | 140 | 120 | 3 | 56 |
| 5 | Ethanolamine | 170 | 120 | 3 | 100 |

^a^ The reaction was carried out with 2 g Kapton film and 20 mL amine at different conditions.

^b^ The degradation rate of Kapton film was calculated according to the equation.

Degradation rate (%) =$\frac{w_{0}-w_{1}}{w_{0}}$× 100%

Where w_0_ and w_1_ are the weight of Kapton film before and after aminolysis, respectively.

**Table S2** Recycling monomers from Kapton film. All the yields are isolated yield.


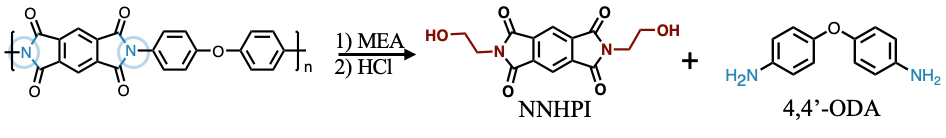


| **Entry** | **Aminolysis**  **Temperature**  **(℃)** | **Aminolysis**  **time**  **(h)** | **Hydrolysis temperature (℃)** | **Hydrolysis**  **time**  **(h)** | **Yield of NNHPI**  **(%)** | **Yield of**  **4,4’-ODA**  **(%)** |
| --- | --- | --- | --- | --- | --- | --- |
| 1 **^a^** | 120 | 1 | 100 | 1 | 28 | 39 |
| 2 **^a^** | 120 | 2 | 100 | 1 | 70 | 80 |
| 3 **^a^** | 120 | 3 | 100 | 1 | 82 | 91 |
| 4 **^a^** | 120 | 5 | 100 | 1 | 74 | 85 |
| 5 **^a^** | 120 | 3 | 90 | 1 | 63 | 90 |
| 6 **^a^** | 120 | 3 | 75 | 1 | 42 | 92 |
| 7 **^a^** | 120 | 3 | 60 | 1 | 7 | 91 |
| 8 **^a^** | 120 | 3 | 25 | 1 | 0 | 96 |
| 9 **^a^** | 120 | 3 | 100 | 2 | 72 | 84 |
| 10 **^a^** | 120 | 3 | 100 | 3 | 70 | 73 |
| 11 **^a^** | 100 | 3 | 100 | 1 | 24 | 34 |
| 12 **^a^** | 140 | 3 | 100 | 1 | 75 | 89 |
| 13 **^b^** | 120 | 5 | 100 | 1 | 76 | 91 |

^a^ 2 g Kapton film and 20 mL MEA were added into a round-bottom flask, and the reaction was performed at different temperatures and times. The aminolysis products were concentrated by distillation under reduced pressure. Then, 15 mL 2M HCl was added and reacted with the aminolysis products at different temperatures and times. After that, NNHPI was collected by filtration. The 4,4’-ODA was precipitated from the filtrate after adding NaOH to alkaline.

^b^ 2 g Kapton film and 20 mL MEA were added into a round-bottom flask, and the reaction was performed at 120 ℃ for 5 h under N_2_. The aminolysis products were concentrated by distillation under reduced pressure. Then, 15 mL 2M HCl was added and reacted with the aminolysis products at 100 ℃ for 1 h under N_2_. After that, NNHPI was collected by filtration. The 4,4’-ODA was precipitated from the filtrate after adding NaOH to alkaline.

**Table S3** Comparison of monomer yields under different hydrolysis conditions.

| **Entry** | **Hydrolysis**  **condition** | **Yield of NNHPI**  **(%)** | **Yield of diamide (%)** |
| --- | --- | --- | --- |
| 1 | 2M HCl | 82 | 91 |
| 2 | 2M NaOH | 0 | 93 |
| 3 | H_2_O | 0 | 88 |

**Table S4** Chemicals recycled from thermosetting PI. All yields are isolated yields ^a^.

| **Entry** | **Aminolysis time**  **(h)** | **Hydrolysis temperature**  **(℃)** | **Hydrolysis time**  **(h)** | **HCl**  **concentration (M)** | **Yield of diamide (%)** |
| --- | --- | --- | --- | --- | --- |
| 1 | 0.5 | 100 | 1 | 2 | 36 |
| 2 | 1 | 100 | 1 | 2 | 49 |
| 3 | 3 | 100 | 1 | 2 | 52 |
| 4 | 6 | 100 | 1 | 2 | 57 |
| 5 | 1 | 25 | 1 | 2 | 25 |
| 6 | 1 | 50 | 1 | 2 | 43 |
| 7 | 1 | 75 | 1 | 2 | 48 |
| 8 | 1 | 100 | 0.5 | 2 | 36 |
| 9 | 1 | 100 | 2 | 2 | 57 |
| 10 | 1 | 100 | 3 | 2 | 61 |
| 11 | 1 | 100 | 6 | 2 | 60 |
| 12 | 1 | 100 | 1 | 0 | 0 |
| 13 | 1 | 100 | 1 | 0.5 | 23 |
| 14 | 1 | 100 | 1 | 1 | 39 |
| 15 | 1 | 100 | 1 | 4 | 53 |

^a^ 2 g PETI and 20 mL MEA were added into a round-bottom flask equipped with a magnetic stir bar. Subsequently, the reaction was performed at 120 ℃ for 0.5-6 h. The resulting mixture was concentrated by distillation under reduced pressure. Then, 15 mL HCl was added and reacted with the mixture at 25-100 ℃ for 0.5-6 h. After that, DPETI was collected by filtration. The diamines were precipitated from the filtrate after adding NaOH to alkaline.

**Table S5** The GPC data of DPETI**.**

| **Sample** | **Mn**  **(Daltons)** | **Mw**  **(Daltons)** | **Mp**  **(Daltons)** | **Mz**  **(Daltons)** | **M_Z+1_**  **(Daltons)** | **Polydispersity** |
| --- | --- | --- | --- | --- | --- | --- |
| DPETI | 969 | 1928 | 1010 | 4841 | 10145 | 1.99 |

**Table S6** TGA data of PET and PET-DPETI-n%.

| **samples** | **N_2_ atmosphere** | | |  | **Air atmosphere** | | | |
| --- | --- | --- | --- | --- | --- | --- | --- | --- |
|  | **T_5%_**  **(℃)** | **T_max_**  **(℃)** | **CR**  **(%)** |  | **T_5%_**  **(℃)** | **T_max1_**  **(℃)** | **T_max1_**  **(℃)** | **CR**  **(%)** |
| PET | 383 | 427 | 14.9 |  | 377 | 418 | 523 | 0 |
| PET-DPETI-2% | 386 | 428 | 16.8 |  | 381 | 418 | 520 | 0 |
| PET-DPETI-4% | 378 | 425 | 18.1 |  | 379 | 418 | 527 | 0 |
| PET-DPETI-6% | 386 | 423 | 19.7 |  | 381 | 429 | 523 | 0 |
| PET-DPETI-8% | 384 | 427 | 18.8 |  | 381 | 427 | 521 | 0 |

**Table S7** GPC date of PET and PET-DPETI-n%.

| **samples** | **Mn** | **Mw** | **PDI** |
| --- | --- | --- | --- |
| PET | 18577 | 34517 | 1.86 |
| PET-DPETI-2% | 14215 | 28513 | 2.00 |
| PET-DPETI-4% | 18673 | 31085 | 1.66 |
| PET-DPETI-6% | 26600 | 32352 | 1.21 |
| PET-DPETI-8% | 13247 | 21935 | 1.66 |

**Table S8** Environmental impact assessment of Kapton chemical recycling using CML2001 method—all scenarios: AP: acidification potential, GWP: global warming potential-100 years, FAETP: freshwater aquatic ecotoxicity potential, MAETP: marine aquatic ecotoxicity potential, TETP: terrestrial ecotoxicity potential, FADP: abiotic depletion-fossil fuels, EP: eutrophication potential, HTTP: human toxicity, EADP: abiotic depletion -elements, ODP: ozone layer depletion potential, steady state. OFP: photochemical oxidation (high NO)

|  | **Materials** | **Amount of material** | **AP** | **GWP** | **FAETP** | **MAETP** | **TETP** | **FADP** | **EP** | **HTP** | **EADP** | **ODP** | **OFP** |
| --- | --- | --- | --- | --- | --- | --- | --- | --- | --- | --- | --- | --- | --- |
| **Unit** | **/** | / | kg  SO_2_ | kg CO_2_ | kg 1,4-DCB | kg 1,4-DCB | kg 1,4-DCB | MJ | kg PO^4-^ | kg 1,4-DCB | kg  Sb | kg CFC-11 | kg ethylene |
| **Input** | **MEA** | 0.700  kg | 0.0072 | 2.2158 | 0.8016 | 1703 | 0.0116 | 0.0108 | 45.97 | 1.24E-05 | 1.90E-08 | 5.53E-04 | 1.24E-05 |
|  | **HCl** | 0.365  kg | 0.0019 | 0.3024 | 0.3071 | 617 | 0.0043 | 0.0018 | 0.76 | 7.42E-06 | 9.69E-08 | 1.23E-04 | 7.42E-06 |
|  | **H_2_O** | 5.035  kg | 0.0000 | 0.0023 | 0.0032 | 5 | 0.0000 | 0.0000 | 0.01 | 8.65E-08 | 1.13E-09 | 1.01E-06 | 8.65E-08 |
|  | **NaOH** | 0.192  kg | 0.0012 | 0.2460 | 0.1571 | 413 | 0.0023 | 0.0005 | 0.35 | 2.25E-06 | 1.44E-07 | 6.58E-05 | 2.25E-06 |
|  | **Heat** | 14.544 MJ | 0.0004 | 0.5502 | 0.0349 | 51 | 0.0007 | 0.0001 | 0.09 | 3.13E-07 | 7.76E-09 | 5.90E-05 | 3.13E-07 |
|  | **Electricity** | 0.004 kwh | 0.0000 | 0.0038 | 0.0019 | 6 | 0.0001 | 0.0001 | 0.00 | 1.59E-08 | 8.23E-12 | 9.71E-07 | 1.59E-08 |
| **Output** | **4,4’-ODA** | 0.485  kg | 0.0991 | 5.4958 | 3.2265 | 7243 | 0.0516 | 0.0227 | 41.93 | 6.09E-05 | 9.09E-07 | 7.00E-03 | 6.09E-05 |
|  | **NNHPI** | 0.6085 kg | 0.0037 | 1.2308 | 0.3182 | 699 | 0.0087 | 0.0039 | 16.18 | 4.58E-06 | 1.19E-08 | 2.99E-04 | 4.58E-06 |

**Table S9** The carbon emission of traditional hydrolysis of Kapton film.

|  | **Materials** | **Amount of material** | **Units** | **kg CO_2_ eq.** |
| --- | --- | --- | --- | --- |
| **Inputs** | NaOH | 0.8333 | Kg | 1.0666 |
|  | H_2_O | 4.1864 | Kg | 0.0020 |
|  | H_2_SO_4_ | 1.7778 | Kg | 1.6711 |
|  | heat | 21.7501 | MJ | 0.7747 |
|  | Electricity | 0.0004 | kwh | 0.0038 |
| **Outputs** | 4,4’-ODA | 0.4417 | Kg | 5.0051 |
|  | PMDA | 0.4542 | Kg | 0.4758 |

**Supplementary references**

[1] J. E. A. Webb, M. J. Crossley, P. Turner, P. Thordarson, J. Am. Chem. Soc. **2007**, 129 (22), 7155.

[2] M. Ding, Polyimides: Chemistry, Structure-Properties Relationships and Materials. The Second Edition, Science Press, **2017**.

[3] J. Guinée, Int. J. Life Cycle. Ass. **2001**, 6 (5), 255.

[4] M. Raimbault, ISO Focus **2006**, (6), 3.

[5] M. R. J. US2576625(A), **1947**.

[6] F. L. C. US2756261(A), **1954**.

[7] G. Wernet, C. Bauer, B. Steubing, J. Reinhard, E. Moreno-Ruiz, B. Weidema, Int. J. Life Cycle Ass. **2016**, 21 (9), 1218.

[8] C. Lee, W. Yang, R. G. Parr, Physical Review B **1988**, 37 (2), 785.

[9] A. D. Becke, Physical Review A **1988**, 38 (6), 3098.

[10] Becke, D. Axel, J. Chem. Phys. **1993**, 98 (7), 5648.
